# The m^6^A-suppressed P2RX6 activation promotes renal cancer cells migration and invasion through ATP-induced Ca^2+^ influx modulating ERK1/2 phosphorylation and MMP9 signaling pathway

**DOI:** 10.1186/s13046-019-1223-y

**Published:** 2019-06-03

**Authors:** Dongkui Gong, Jin Zhang, Yonghui Chen, Yunfei Xu, Junjie Ma, Guanghui Hu, Yiran Huang, Junhua Zheng, Wei Zhai, Wei Xue

**Affiliations:** 1 Department of Urology, Shanghai Tenth People’s Hospital, School of Medicine in Tongji University, Shanghai, 200072 China; 2 Department of Urology, Renji Hospital, School of Medicine in Shanghai Jiao Tong University, Shanghai, 200127 China; 3 Department of Urology, Pudong Hospital, School of Medicine in Fudan University, Shanghai, 201300 China; 4 Department of Urology, Shanghai First People’s Hospital, School of Medicine in Shanghai Jiao Tong University, Shanghai, 200080 China

**Keywords:** Renal cell carcinoma, P2RX6, m^6^A, Ca^2+^, p-ERK1/2/MMP9

## Abstract

**Background:**

Previous study demonstrated that extracellular ATP could promote cell migration and invasion in multiple human cancers. Till now, the pro-invasive mechanisms of ATP and P2RX6, a preferred receptor for ATP, are still poorly studied in RCC.

**Methods:**

Bioinformatics analysis was performed to identify the differentially expressed genes during RCC different stages. Tissue microarray, IHC staining and survival analysis was respectively used to evaluate potential clinical function. In vitro and in vivo assays were performed to explore the P2RX6 biological effects in RCC progression.

**Results:**

We found that ATP might increase RCC cells migration and invasion through P2RX6. Mechanism dissection revealed that ATP-P2RX6 might modulate the Ca^2+^-mediated p-ERK1/2/MMP9 signaling to increase the RCC cells migration and invasion. Furthermore, METTL14 implicated m^6^A modification in RCC and down-regulated P2RX6 protein translation. In addition, human clinical survey also indicated the positive correlation of this newly identified signaling in RCC progression and prognosis.

**Conclusions:**

Our findings revealed that the newly identified ATP-P2RX6-Ca^2+^-p-ERK1/2-MMP9 signaling facilitates RCC cell invasion and metastasis. Targeting this novel signaling pathway with small molecules might help us to develop a new approach to better suppress RCC progression.

**Electronic supplementary material:**

The online version of this article (10.1186/s13046-019-1223-y) contains supplementary material, which is available to authorized users.

## Background

Renal cell carcinoma (RCC) is the most lethal of all urological malignancies, accounting for about 3.8% of all new malignancies, with about 65,340 new cases and 14,970 deaths estimated for 2018 in the United States alone [1, 2] . Situation in other countries is also roughly the same [3]. However, RCC is basically radiotherapy and chemotherapy resistant and best treated by resection [4–6]. Although tyrosine kinase inhibitor (TKI)-based antiangiogenic therapy is the standard treatment for metastatic RCC (mRCC) and has improved patients’ prognosis, but the effect is still limited due to drug resistance [7, 8]. Thus, it is urgent to elucidate the underlying mechanisms of RCC earlier metastasis and find out more clinical indicator and therapeutic target for RCC metastasis.

Adenosine 5′-triphosphate (ATP) is the main form of intracellular energy for all types of cell and played different roles in multiple cancers development [9, 10]. It is also released extracellular, both in physiological and pathological conditions, where it mediates various biological functions via activation of P2 receptors [11–13]. The P2 receptors have two subfamilies in mammalian cells, P2X and P2Y. P2X family of ligand-gated ion channel receptors have seven members, P2RX1–7, which all constitute non-selective cation channels proposed to be functional under a homo- or heterotrimeric association [11, 14]. P2Y family of G protein-coupled receptors, including P2RY1, 2, 4, 6, 11, 12, 13, 14 [15, 16], which can modulate the adenylate cyclase-cyclic adenosine 3′, 5′-monophosphate pathway or stimulate the phospholipase C (PLC)-Ca^2+^ signaling pathway [17].

Previous reports revealed that prinergic receptor subtypes were involved in the development of multiple tumors, such as prostate, bladder, breast et al. [9, 10, 18]. It is demonstrated that P2RY1 and P2RY2 receptors were expressed and involved in prostate cancer cell proliferation; P2RX5 receptors were involved in cell differentiation and P2RX7 receptors were involved in cell death. In human squamous cell carcinoma, P2RY2, P2RX5 and P2RX7 seem to correlate with cell proliferation, differentiation and cell death, respectively [19]. Here, we found that P2RX6, a preferred receptor for ATP, contributed to the invasion and metastasis of RCC cells. However, the potential signaling pathway, especially in RCC metastasis, is still not clear.

N6-methyladenosine (m^6^A), a predominant internal modification of RNA in higher eukaryotes, aroused people’s attention. This mechanism involves two important catalytic proteins, demethylase and methyltransferase [20]. Recently, numbers of studied showed that m^6^A modification played a crucial role in multiple tumor development [21–23], such as nuclear RNA export, transcription splicing, cell fate determination and protein translation control [24–26]. Previous study revealed that methyltransferase-like 14 (METTL14) could suppress the metastatic potential of hepatocellular carcinoma by modulating m^6^A dependent primary microRNA processing [22]. In addition, hypoxia could induce the breast cancer stem cell phenotype by HIF-dependent and ALKBH5-mediated m^6^A of NANOG mRNA [23]. However, the status of m^6^A modification and the underlying regulatory mechanisms in RCC remained incompletely understood.

## Materials and methods

### Bioinformatics analysis

We obtained the KIRC patient data from the TCGA database collection (https://xenabrowser.net/datapages/). The database is generated by the TCGA Research Network: http://cancergenome.nih.gov/. GO pathway analysis was obtained from http://geneontology.org/. Gene expression and survival data were got from http://ualcan.path.uab.edu/. The demographic information such as gender, age, BMI and tumor clinical characteristics such as gene expression, OS and TNM status were collected. Clinical TNM staging were based on the UICC 8th edition [27] and we excluded patients without TNM stage information from the analysis.

### Cell lines and chemicals

The OS-RC-2, 786-O, and HEK-293 cells were obtained from Cell Bank of the Chinese Academy of Sciences (Shanghai, China). SN12-PM6 cell line was kindly provided by Dr. Qingbo Huang from the Department of Urology, Chinese PLA General Hospital, Beijing, China. SW839 cell line was kindly provided by Dr. Chawnshang Chang from George Whipple Lab for Cancer Research, University of Rochester Medical Center, Rochester, NY, 14646 USA. All cell lines were expanded to passage 3, stored in aliquots in liquid nitrogen, and were used for fewer than 4 months after receipt or resuscitation from cryopreservation. Dulbecco’s Modified Eagle’s Medium (DMEM, Gibco, USA) was used to culture cells above. All cells described above were cultured at 37 °C in 5% CO_2_ and tested for mycoplasma every 6 months using the Universal Mycoplasma Detection Kit (ATCC, USA).

### Lentivirus packaging

The standard calcium chloride transfection method was performed. The pMD2G envelope plasmid and psAX2 packaging plasmid, with interest gene knockdown/ overexpression plasmid were transfected into HEK293T cells for 48 h to generate the lentivirus supernatant, oligo sequences were listed in Additional file 1: Table S1. The lentivirus supernatants were harvest through a 0.45 μm nitrocellulose filter and used immediately or frozen in − 80 °C for later use.

### Wound healing migration assay

Cells were seeded into six-well plates at a density that after 24 h of growth, they reached approximately 60% confluence. Gently and slowly scrape the single layer with a new pipette tip across the center of the well. Then, washed wells twice with PBS to remove the detached cells and replenished with fresh medium. The cells were grown for an additional 24 h. Images were captured of the monolayer using a microscope. The gap distance was quantitatively evaluated using ImageJ v1.51 (Wayne Rasband, USA). The relative migration was determined by setting the number of average migration distance in control group as one. The experiments were tested in triplicate.

### Transwell invasion assay

Cell invasion capacity was assessed by a matrigel invasion assay using matrigel coating 8.0 μm filter membranes. Cells (5 × 10^4^) in 150 μL of serum-free medium were plated onto each filter, with 600 μL of 10% serum-containing medium placed in the lower chamber and then incubated for 12 h in cell culture incubator at 37 °C in a 5% CO_2_ atmosphere. After 12 h, removed the cells on the upper surface of the filters with cotton swabs. Filters were fixed with 4% paraformaldehyde in both sides and washed with PBS, and stained with crystal violet. Cells that had invaded to the lower surface of the filter were counted with Image-pro Plus v6.0 (Media Cybernetics, USA) in 5 randomly selected fields. The relative invasion was determined by setting the number of invading cells in control group as one. The experiments were tested in triplicate.

### RNA extraction and quantitative real-time PCR analysis (qRT-PCR)

Trizol reagent (Invitrogen, USA) was used to extract the total RNA from cells or tissues according to the manufacturer’s protocol. PrimeScript RT reagent Kit (Takara, Japan) was used to synthesize cDNAs. RT-qPCR was performed with KAKA SYBR FAST qPCR Kit (Kapa Biosystems, USA) using a 7900HT Fast Real-Time PCR System (Applied Biosystems, Japan). All primers were listed in Additional file 1: Table S1. The expression level was normalized to endogenous small nuclear RNA U6 or GAPDH. The 2^−ΔΔCt^ method was used to analyze the expression level relative to the endogenous control.

### Western immunoblotting analysis (WB)

Cells were lysed on ice using RIPA buffer plus phosphatase inhibitors and protease inhibitors. For western blot analysis, 25 μg of protein extracts were loaded to 10% sodium dodecyl sulfate-polyacrylamide gel electrophoresis gels and transferred to nitrocellulose membranes. Primary antibody was used to incubate the membranes at 4 °C overnight and secondary antibody for 1 h with room temperature. GAPDH or Actin expression were used as loading control. The antibodies used were listed in Additional file 1: Table S1.

### Immunofluorescence assay (IF)

Cells were grown on coverslips and fixed in 4% paraformaldehyde at room temperature for 10 min. After PBS washing, the cells were blocked with 3% BSA at 37 °C for 30 min and incubated at 4 °C with anti-p-ERK1/2 or MMP9 overnight, and then probed with a tetramethylrhodamine isothiocyanate (TRITC)-conjugated secondary antibody (Sigma, USA) at 37 °C for 2 h. Subsequently, cells were stained with DAPI and observed under a fluorescence microscope.

### Measurement of intracellular calcium ion concentration

We measured the calcium ion ((Ca^2+^)i) concentration according to the method in the published papers [28, 29]. Briefly, cells were stained with 5 μM Fluo-3 AM and washed with physiological solution (125 mM NaCl, 5 mM KCl, 1 mM MgCl_2_, 10 mM HEPES, 5 mM glucose, and 1 mM CaCl_2_). Then, cells were treated with ATP and fluorescent images were scanned every 5 s using a confocal microscope (IX70 Fluoview, Olympus, Japan; excitation wavelength 488 nm, emission wavelength 530 nm). The changes in (Ca^2+^)i were calculated as follows: Change in (Ca^2+^)i = (F_max_-F_0_) / F_0_**.** F, the fluorescence intensity; F_0_, the basal fluorescence intensity before treatment; F_max_, the maximum level of fluorescence intensity.

### m^6^A quantification assay

The m^6^A content in the total RNAs were measured with the m^6^A RNA methylation quantification kit (ab185912, Abcam, UK). Briefly, 200 ng RNAs were coated on assay wells. According to the manufacturer’s instructions, detection antibody solution and capture antibody solution were then added to assay wells separately in a suitable diluted concentration. Reading the absorbance at 450 nm and quantifying the m^6^A levels. Statistical analysis were performed based on the standard curve.

### Mouse tail vein injection xenograft models

For the in vivo metastasis assays, luciferase labeled OS-RC-2 cells stably expressing OE-P2RX6 or pWPI-vector were injected into the tail vein of 5 weeks old BALB/c nude mice (Sipper-BK laboratory animal Company, Shanghai, China). All mice were maintained in an SPF level environment and were manipulated according to protocols approved by the Institutional Animal Care and Use Committee of the Shanghai Tenth People’s hospital. Each tumor cell sub-line was injected into ten mice (2 X 10^6^ per mouse). IVIS Lumina imaging system (Calipers, Hopkinton, USA) was used to observe tumor metastasis. After 8 weeks, sacrificed the mice, harvested the tissues and fixed in 10% neutral PB-buffered formalin (pH 7.4). The fixed samples were then embedded in paraffin. The sections were stained with H&E and antibody for analyzing the presence of metastases.

### Immunohistochemical staining (IHC)

IHC was performed on the samples from the human RCC tissues and mouse xenografted tumors, as described previously [30]. Briefly, the samples were fixed in 4% neutral buffered paraformaldehyde and embedded in paraffin and cut into 5 μm slices. After deparaffnization, hydration, and antigen retrieval, these sections were incubated with corresponding primary antibodies, incubated with biotinylated secondary antibodies (Vector Laboratories, Burlingame, CA, USA) and then visualized by VECTASTAIN ABC peroxidase system and 3, 3′-diaminobenzidine (DAB) kit (Vector Laboratories). The slides were scored by two experienced pathologist without the knowledge of patient outcome. The expression was assessed semi-quantitatively as follows: negative (−) < 5%, 5–25% (+, weak positive), 25–50% (++, moderate positive) and > 50% (+++, strong positive). Negative and weak positive expressions were defined as low expression, while positive and strong positive expressions were defined as high expression. Image-pro Plus v6.0 (Media Cybernetics, USA) was used to quantify the integral optical density (ISO) in 5 randomly selected fields.

### Clinical samples

A total of 17 RCC samples and paired para-tumor tissues were obtained for patients who underwent partial or radical nephrectomy in Department of Urology, Shanghai Tenth People’s Hospital, Tongji University (Shanghai, China). The 238 specimens which were included in tissue microarray, Kaplan-Meier survival analysis and Cox regression analysis were obtained from Department of Urology database, these samples had corresponding at least 5 years follow-up information. The fresh tissues were frozen in liquid nitrogen to protect the protein and RNA away from degradation. The use of human tissues was approved by the ethics committee of Shanghai Tenth People’s Hospital.

### Statistical analysis

All data were analyzed using the Graphpad 7.0 (GraphPad Software, USA) and SPSS 23 (IBM, USA). Results are expressed as Means ± S.D. from at least 3 independent experiments. Student’s T Test was used for comparing two groups data and one-way ANOVA followed by individual comparisons with Dunnett’s test was use for comparing more than two groups data. All data was considered significantly when *P* < 0.05.

## Results

### P2RX6 is highly expressed and associated with poor prognosis of RCC through TCGA database

**Fig. 1:**
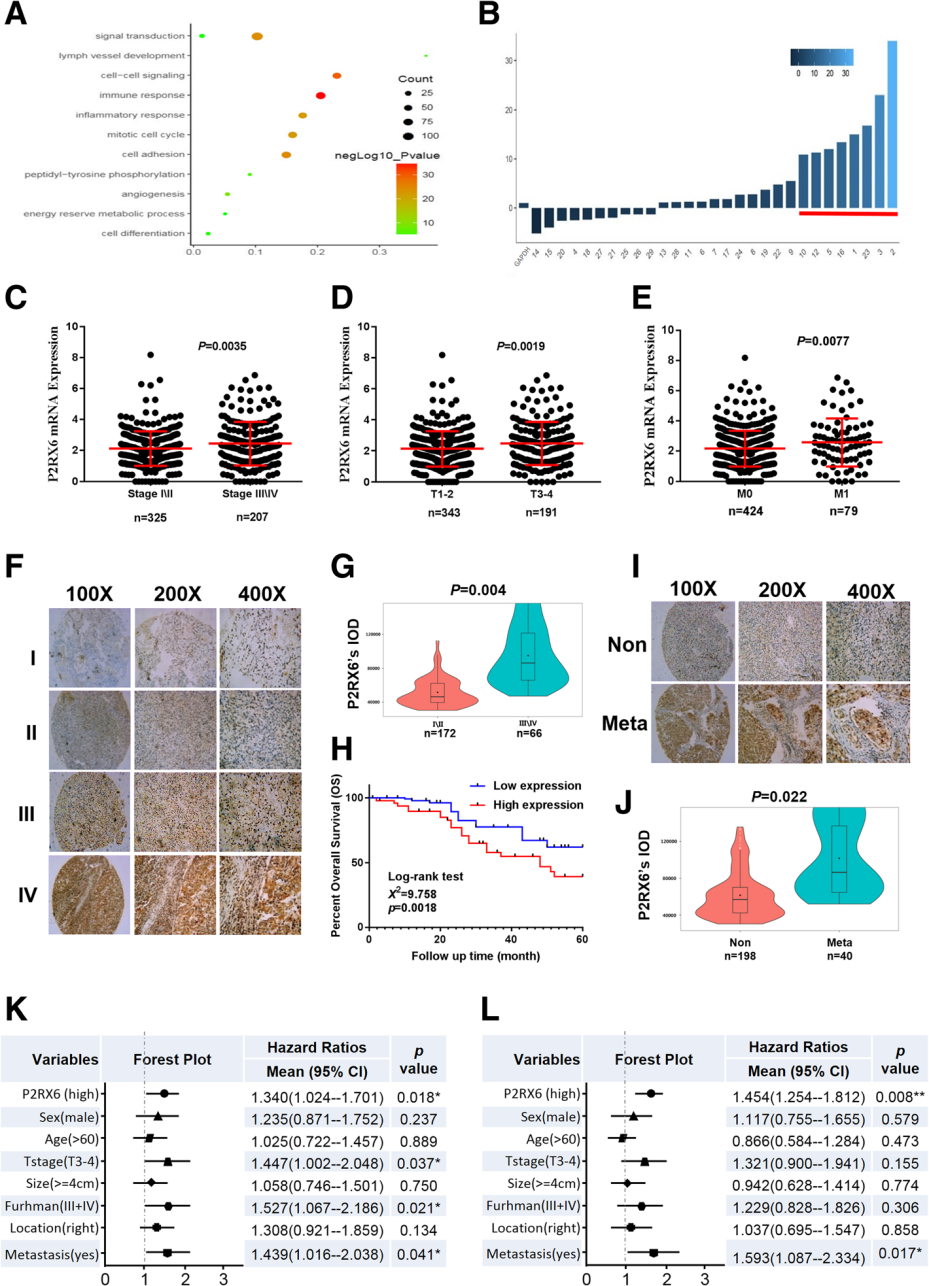
P2RX6 is highly expressed and associated with RCC poor prognosis. **a** 628 DEGs’ Go pathway enrichment analysis. **b** qRT-PCR assay validation of 29 DEGs mRNA expression in 10 paired clinical tumor compared with para-tumor samples. **c** TCGA database analysis revealed P2RX6 expression is higher in stage III and stage IV (*n* = 207) compared with stage I and stage II (*n* = 325) (**P = 0.0035). **d** TCGA database analysis revealed P2RX6 expression is higher in T3 and T4 (*n* = 191) compared with T1 and T2 (*n* = 343) (**P = 0.0019). **e** TCGA database analysis revealed P2RX6 expression is higher in M1 (*n* = 79) compared with M0 (*n* = 424) (**P = 0.0077). (F-L) Human clinical studies for P2RX6 expression in RCC patients. **f** Human clinical samples analysis revealed P2RX6 expression is higher in higher RCC pathological stage samples. **g** Violin plot quantitative analysis for Fig. 1f. **h** Kaplan-Meier analysis for different P2RX6 protein expression in RCC patients (**P = 0.0018). **i** Representative IHC image of P2RX6 expression in Non and Meta group tissue samples. **j** Violin plot quantitative analysis for Fig. 1i. **k** Univariate Cox regression analysis and forest map. **l** Multivariate cox regression analyses and forest map. Non means non-metastatic group, and Meta means metastatic group. * *P* < 0.05, ** *P* < 0.01

The candidates selecting process was described as Additional file 8: Figure S1 A. We downloaded 534 samples clinical information from TCGA database (Additional file 2: Table S2) and got 628 differentially expressed genes (DEGs) with the screening criteria G4/G1 > 3 and *P* < 0.001. Validations of 44 DEGs were got by intersected with GO pathway analysis (Fig. 1a) top six signaling pathway. Meanwhile, 29 DEGs were consistent with their survival outcome. Furthermore, we used qRT-PCR in 10 paired clinical renal tumor and para-tumors samples and revealed that 8 DEGs increased in renal tumor samples significantly (Fig. 1b). Specific information in the screening process and GO analysis result can be seen in Additional file 3: Table S3. Among the remaining 8 DEGs (ADRA2A, P2RX6, XCL2, XCL1, CCL7, TRIM54, TNFRSF18 and SPOCK1), the expression of ADRA2A, the top one, in KIRC based on individual cancer is lower than the normal tissues (Additional file 8: Figure S1 B), but patients with lower expressed ADRA2A have a better overall survival (OS) (Additional file 8: Fig. S1 C). On the other hand, P2RX6 had the dramatically differential expression between normal and tumor tissues in our clinical samples (Fig. 1b). Meanwhile, TCGA clinicopathologic correlation analysis suggested that P2RX6 expression associated with RCC pathological stage, pathological grade, metastasis (**P = 0.0035, 0.0019, 0.0077, respectively) (Fig. 1c-e). In addition, P2RX6 mRNA expression correlated with RCC patients OS, indicating higher expression of P2RX6 associated with RCC poor prognosis (**P = 0.00175) (Addtional file 8: Figure S1 D). Meanwhile, subsequent loss-of-functional experiments also confirmed that P2RX6 might be the key candidate what we were looking for.

Together, results from Fig. 1a-e and Additional file 8: Figure S1 A-D suggested that P2RX6 might be involved in RCC progression and function as a potential biomarker in prognostic value.

### Human clinical studies for P2RX6 expression in RCC patients

Next, to link the analysis result from TCGA RCC database, we analyzed IHC staining results from 238 human RCC samples which revealed the median number of P2RX6 expression was IOD 61725.5. Clinicopathologic correlation analysis proved that P2RX6 expression associated with RCC pathological stage (**P = 0.004) (Fig. 1f-g, Additional file 4: Table S4). In addition, P2RX6 protein expression correlated with RCC patients OS, indicating higher expression of P2RX6 associated with RCC poor prognosis (**P = 0.0018) (Fig. 1h). Besides, P2RX6 was also associated with RCC metastasis (*P = 0.022) (Fig. 1i-j, Additional file 4: Table S4).

In addition, we explored the correlation between P2RX6 and RCC clinical pathological characteristics. Correlation regression analysis of 238 samples demonstrated that high expression of P2RX6 was clearly associated with T stage (**P = 0.004), Fuhrman grade (*P = 0.039) and metastasis (*P = 0.022) (Additional file 5: Table S5). Meanwhile, we implemented univariate and multivariate cox regression models to analyze the correlation of the multiple variables with OS of 238 RCC patients. Univariate analysis demonstrated that a higher level of P2RX6 (hazard ratio, HR = 1.340; 95% confidence interval, CI = 1.024--1.701; *P = 0.018), a higher T stage (HR = 1.447; 95% CI = 1.002--2.048; *P = 0.037), higher Furhman grade (HR = 1.527; 95% CI = 1.067--2.186; *P = 0.021) and more metastasis (HR = 1.439; 95% CI = 1.016--2.038; *P = 0.041) were potently correlated with OS (Fig. 1k). Multivariate analysis substantiated that a higher P2RX6 expression level (HR = 1.454; 95% CI = 1.254--1.812; **P = 0.008) and more metastasis (HR = 1.593; 95% CI = 1.087--2.334; *P = 0.017) were markedly associated with OS (Fig. 1l).

Taken together, these results indicated that RCC patients’ samples are in agreement with online database suggesting P2RX6 was higher expressed in RCC tissues than normal renal tissues and correlated with poor prognosis.

### P2RX6 promotes RCC cell migration and invasion in vitro

**Fig. 2:**
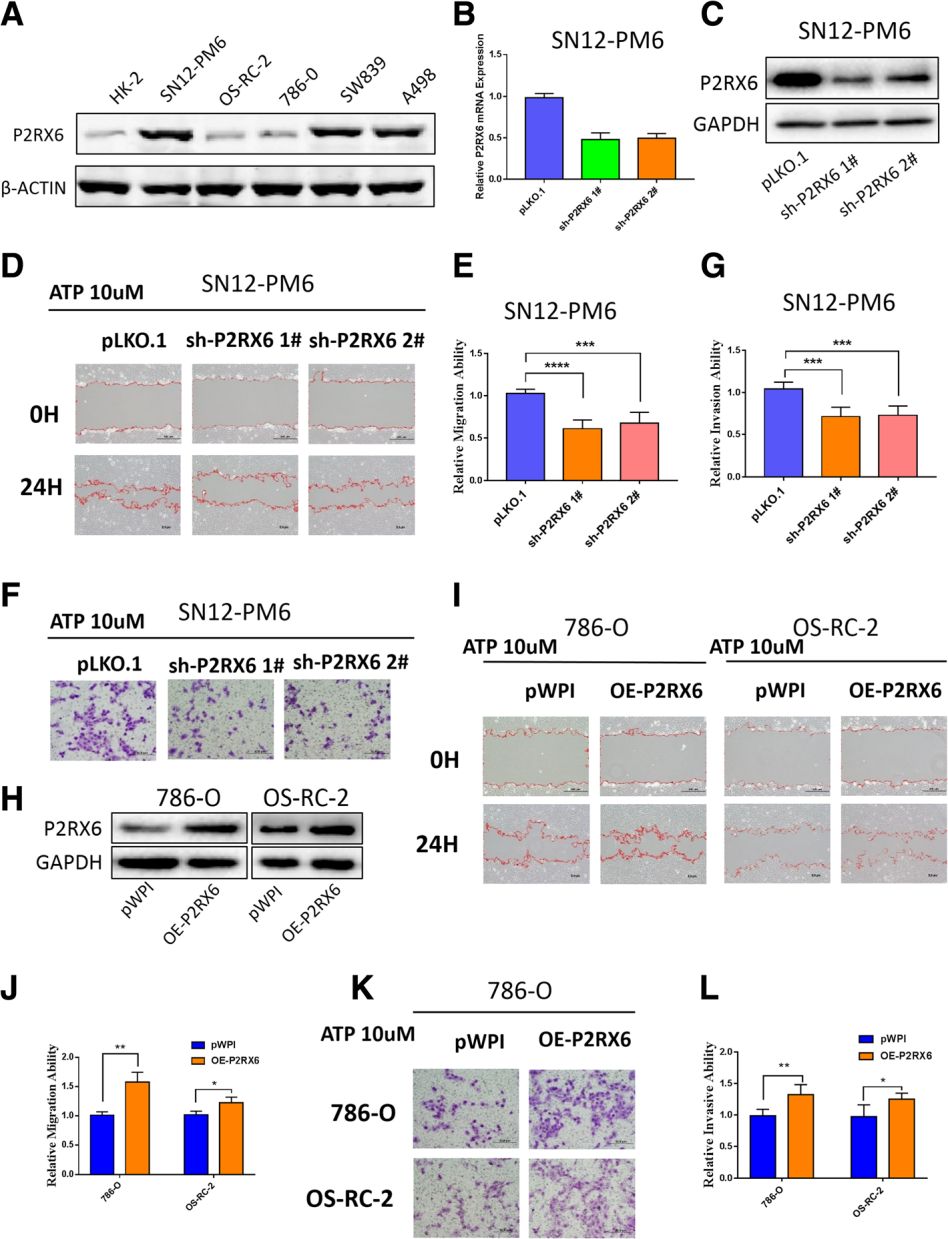
P2RX6 mediated the ATP-enhanced RCC migration/invasion. **a** WB validation of P2RX6 protein expression was elevated in multiple RCC (SN12-PM6, OS-RC-2, 786-O, SW839, A498) vs. normal renal proximal tubule epithelial cell line HK-2. **b** qRT-PCR assay validation of P2RX6 mRNA level knocking-down efficiency and **c** WB validation of P2RX6 protein level knocking-down efficiency when knocking down using sh-P2RX6^#1^, sh-P2RX6^#2^ in SN12-PM6 cells. **d** Migration assay after using 2 specific P2RX6 shRNAs in SN12-PM6 cells treated with ATP, PLKO.1-vector as control. **e** Quantitative analysis for Fig. 2D. **f** Transwell assay were performed after using 2 specific P2RX6 shRNAs in SN12-PM6 cells treated with ATP, PLKO.1-vector as control. **g** Quantitative analysis for Fig. 2f. **h** WB validated P2RX6 was overexpressed when using P2RX6-cDNA in 786-O and OS-RC-2 cells. **i** Migration assay after P2RX6 overexpression in 786-O and OS-RC-2 cells treated with ATP, pWPI-vector as control. **j** Quantitative analysis for Fig. 2i. **k** Transwell assay after P2RX6 overexpression in 786-O and OS-RC-2cells treated with ATP, PWPI-vector as control. **l** Quantitative analysis for Fig. 2k. * *P* < 0.05, ** *P* < 0.01, *** *P* < 0.001, **** *P* < 0.0001

We first tested P2RX6 protein expression in multiple RCC cell lines (SN12-PM6, OS-RC-2, 786-O, SW839, A498) vs. normal renal proximal tubule epithelial cell line HK-2, and found its expression was elevated in most RCC cell lines (Fig. 2a). Previous study demonstrated that ATP induced breast cancer cells migration and invasion. To investigate ATP effect on RCC, we treated OS-RC-2 cells with different concentration of ATP. Results from “wound-healing” migration assay revealed that 10uM ATP increased a substantial migration of the cells into the scratched area (Additional file 9: Figure S2 A). Similar results were also obtained when we performed transwell invasion assay (Additional file 9: Figure S2 B-C).

Then, we therefore focused on whether P2RX6 played an impressive role in this energy metabolism. Then, we applied the interruption approaches using two specific shRNAs (sh-P2RX6 1# and 2#) to block P2RX6 expression (see their suppressing effects on mRNA level in Fig. 2b and protein level in Fig. 2c), and results revealed that suppressed P2RX6 expression led to block the ATP-increased cell migration (Fig. 2d-e) and invasion (Fig. 2f-g) in SN12-PM6 cells. We also performed the rescue assay with another cell line, SW839, and we found the result was coincident with SN12-PM6’s phenotype (Additional file 9: Figure S2 D-I). Next, we used the opposite approach via adding P2RX6-cDNA (Fig. 2h) into 786-O and OS-RC-2 cell, and results revealed that increased P2RX6 expression conversely led to increase ATP effects on up-regulating cell migration (Fig. 2i-j) and invasion (Fig. 2k-l) in both cells.

Together, results from Fig. 2a-l suggested ATP might function via this newly identified P2RX6 to increase RCC cell migration and invasion.

### Mechanism dissection of how P2RX6 elevates the RCC migration and invasion: via altering Ca^2+^ influx ion channel signaling

Previous studies indicated that parts of P2RX receptors family involved in membrane trafficking, ion permeation (including calcium permeability) and ATP binding [31, 32]. Also, ATP elicited increases in intracellular Ca^2+^ concentration could be repressed by the specific antagonist [33].

**Fig. 3:**
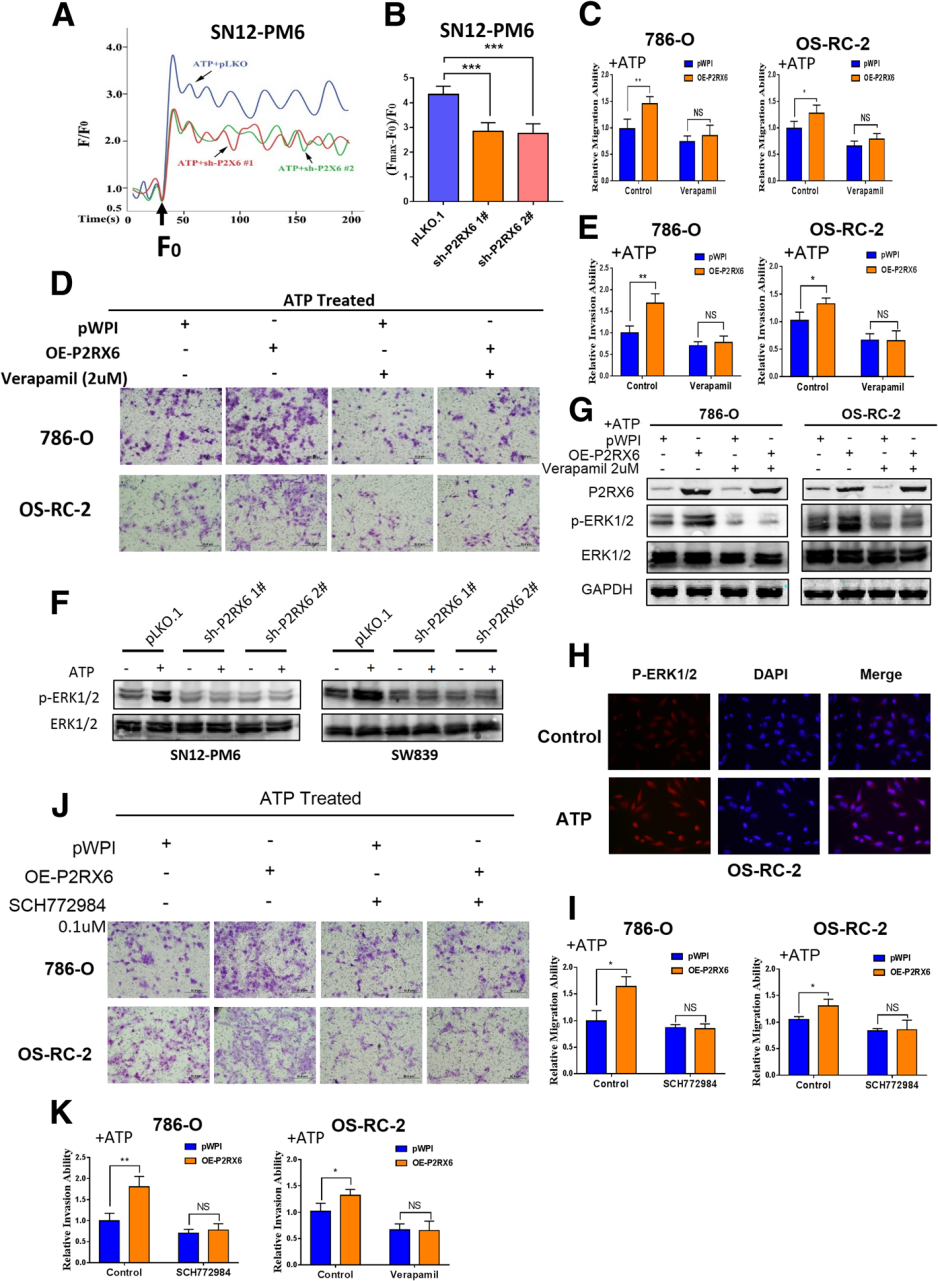
The ATP-P2RX6-Ca^2+^ signaling mediated the MAPK family ERK1/2 activation to promote RCC migration/invasion. **a** Intracellular Ca^2+^ levels were determined in SN12-PM6 to evaluate the P2RX6 effect. Arrows indicate the points at which ATP (10 μM) was added. **b** The net change in Ca^2+^ levels was normalized to (F_max_-F_0_)/F_0_. **c** Migration assay showed Ca^2+^ inhibitor verapamil (2uM) could block ATP-P2RX6 induced cell migration in 786-O and OS-RC-2 cells. **d** Transwell assay showed Ca^2+^ inhibitor verapamil (2uM) could block ATP-P2RX6 induced cell migration in 786-O and OS-RC-2 cells. **e** Quantitative analysis for Fig. 3d. **f** Western Immunoblotting for MAPK family ERK1/2 and p-ERK1/2 expression using sh-P2RX6 1# and 2# treat with/without DHT, GAPDH as an internal control. **g** WB for MAPK family ERK1/2 and p-ERK1/2 expression using verapamil treat with/without over-express P2RX6, GAPDH as an internal control. **h** Immunofluorescence showed that ATP could stimulate the expression of p-ERK1/2 in both the cellular cytoplasm and nucleus. **i** Migration assay showed ERK1/2 inhibitor SCH772984 (0.1uM) could block over-expression P2RX6 induced cell migration in 786-O and OS-RC-2 cells. **j** Transwell assay showed ERK1/2 inhibitor SCH772984 (0.1uM) could block over-expression P2RX6 induced cell invasion in 786-O and OS-RC-2 cells. **k** Quantitative analysis for Fig. 3j. *N.S.* = not significant. * *P* < 0.05, ** *P* < 0.01, *** *P* < 0.001

To further explore P2RX6’s function in RCC, the intracellular Ca^2+^ level (Ca^2+^)i was be measured in response to agonist ATP. 10 μM ATP caused the immediate and rapid increase in (Ca^2+^)i in SN12-PM6, which was significantly decreased in P2RX6-knocked-down group compared to control group (Fig. 3a-b), suggesting P2RX6 participated in the process ATP-induced Ca^2+^ influx activity.

To identify which specific mechanism the ATP-P2RX6 might promote RCC cell migration and invasion, we then applied the interruption approaches with Ca^2+^ specific inhibitor, verapamil, to examine their interruption effects on the ATP-P2RX6-increased cell migration and invasion. The results revealed that adding the 2uM verapamil selectively block Ca^2+^ influx could lead to block ATP-increased cell migration and invasion (Fig. 3c-e) in 786-O and OS-RC-2 cells.

Together, results from Fig. 3a-e using rescue assay concluded that ATP-P2RX6 might function via altering Ca^2+^ influx to increase the RCC cell migration and invasion.

### Mechanism dissection of how ATP-P2RX6 axis can facilitate RCC migration and invasion: via increasing the MAPK family ERK1/2 phosphorylation signaling

Next, in order to explore the intra-cellular downstream signals to mediate the ATP-P2RX6-increased the RCC cell migration and invasion, we focused on the MAPK family ERK1/2 signals since our team recently contributed to this signaling pathway in recent years [34–36]. Meanwhile, early studies indicated the Ca^2+^ influx could effect on the MARK signaling to influence various human cancer cells [37–39].

We first demonstrated that adding 10uM ATP led to activate MARK family ERK1/2 via increasing their phosphorylation in RCC cell lines. We then added P2RX6-shRNA or Ca^2+^ inhibitor verapamil, and results revealed that adding these shRNAs or inhibitor could result in decreasing the phosphorylation of MARK family ERK1/2 (Fig. 3f-g). IF assay demonstrated that ATP treatment up-regulated p-ERK1/2 expression in both cytoplasm and nucleus, indicating that ATP not only stimulates p-ERK1/2 expression but also accelerates its translocation from cytoplasm to nucleus in RCC (Fig. 3h). Meanwhile, adding inhibitor SCH772984 0.1uM specific to ERK1/2 could interrupt the ATP-enhanced RCC migration (Fig. 3i) and invasion (Fig. 3j-k) in both 786-O and OS-RC-2 cells.

Together, results from Fig. 3f-k suggested that ATP-P2RX6-Ca^2+^ axis may function via increasing the MARK family ERK1/2 phosphorylation to enhance RCC cell migration and invasion.

### Mechanism dissection of how ATP-P2RX6-Ca^2+^ −p-ERK1/2 axis augments RCC cell migration and invasion: via promoting MMP9 expression

**Fig. 4:**
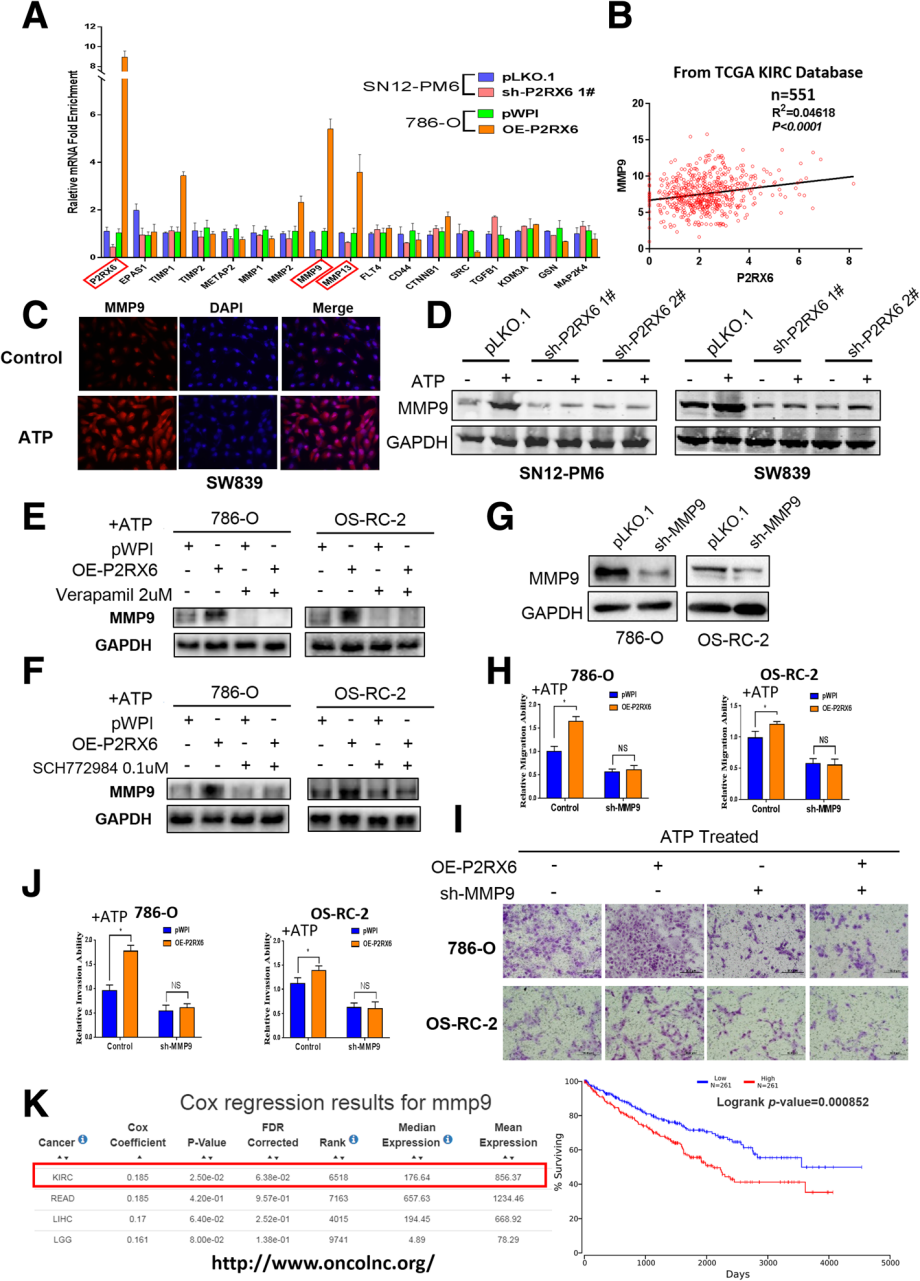
The ATP-P2RX6-Ca^2+^ signaling mediated the MAPK family ERK1/2 activation to promote RCC migration/invasion. **a** qRT-PCR assay for screening some metastasis genes after manipulating P2RX6 in SN12-PM6 and 786-O cells. **b** Correlation analysis for P2RX6 and MMP9 from TCGA database. **c** Immunofluorescence showed that ATP could stimulate the expression of MMP9. **d** WB of MMP9 using P2RX6-shRNA. **e** WB of MMP9 using Ca^2+^ inhibitor verapamil (2uM). **f** WB of MMP9 using ERK1/2 inhibitor SCH772984 (0.1uM). WB to show MMP9 Knockdown efficiency in J82 and 5637 cells. **g** WB of MMP9 using MMP9-shRNA. **h** Migration assay using sh-MMP9 in 786-O and OS-RC-2 cells treated with ATP. **i** Transwell assay after sh-MMP9 in 786-O and OS-RC-2 cells treated with ATP. Scale bar, 200 μm. **j** Quantitative analysis for Fig. 4i. **k** MMP9 gene information on http://www.oncolnc.org/ and Kaplan-Meier analysis for MMP9 mRNA expression in RCC patients (***P = 0.000852). *N.S.* = not significant. * *P* < 0.05, ** *P* < 0.01, *** *P* < 0.001

To search the downstream potential genes that can mediate the ATP-P2RX6-Ca^2+^ −p-ERK1/2-increased RCC cell migration and invasion, we then screen several metastasis related genes that expressed in both SN12-PM6 and 786-O cells, and results revealed that manipulating P2RX6 could produce consistent changes in MMP9 and MMP13 mRNA expression (Fig. 4a). TCGA database indicated that P2RX6 has positive correlation with MMP9 and MMP13 mRNA expression (Fig. 4b, Additional file 10: Figure S3 A). Besides, IF assay previously demonstrated that ATP treatment up-regulated MMP9 expression in RCC (Fig. 4c). WB analyses further confirmed MMP9 protein level was induced after adding 10uM ATP and knocking down P2RX6 could abrogate this effect (Fig. 4d). However, MMP13 failed to obtain this result (Additional file 10: Figure S3 B).

Then we focused on the MMP9 as both its mRNA and protein expressions were all elevated after adding ATP. The interruption approaches via adding the P2RX6-shRNA, Ca^2+^ inhibitor verapamil or ERK1/2 inhibitor SCH772984 all resulted in attenuating MMP9 expression at protein level (Fig. 4d-f). Importantly, adding MMP9-shRNA effectively suppressed MMP9 expression (Fig. 4g), which then resulted in blocking the P2RX6-induced RCC cell migration and invasion in OS-RC-2 and 786-O cells (Fig. 4h-j). In addition, MMP9 mRNA expression correlated with RCC patients OS, indicating higher expression of MMP9 associated with RCC poor prognosis (***P = 0.000852) (Fig. 4k, figure obtained from OncoLnc website http://www.oncolnc.org/).

Together, results from Fig. 4a-k suggested that ATP-P2RX6-Ca^2+^ −p-ERK1/2 axis may function via maximizing the metastasis gene MMP9 expression to facilitate RCC migration and invasion.

### The m6A modification is decreased in RCC and P2RX6 can be regulated by METTL14

**Fig. 5:**
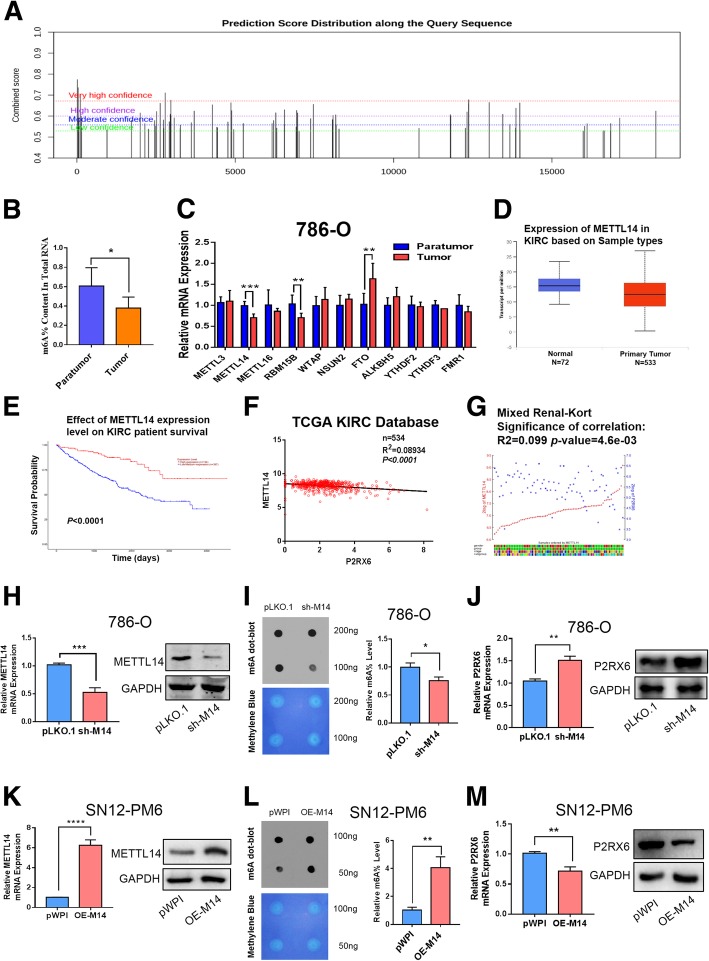
The m^6^A modification is decreased in RCC and P2RX6 can be regulated by METTL14. **a** P2RX6 potential m^6^A sites distribution. **b** The m^6^A contents of total RNAs in tumor tissues paired with para-tumor tissues (n = 7). **c** The mRNA levels of m^6^A modification associated genes in 7 pairs of RCC tumor and para-tumor tissues. **d** Expression of METTL14 in TCGA KIRC. **e** Kaplan-Meier analysis for METTL14 expression level on KIRC patients’ OS. **f** Correlation between METTL14 and P2RX6 from TCGA database. **g** Correlation between METTL14 and P2RX6 from website *R*^2^. **h** Validation of METTL14 mRNA and protein level knocking-down efficiency in 786-O cells. **i** The m6A contents of total RNAs in 786-O. Dot blot assay analyzed the Poly(A)^+^ RNAs isolated from pLKO and sh-M14 group cells. RNAs were equally loaded by two fold serial dilution. Methylene blue was used to detect equal mRNA loading in two groups. **j** P2RX6 mRNA and protein expression level after knocking-down METTL14. **k** Validation of METTL14 mRNA and protein level knocking-down efficiency in OS-RC-2 cells. **l** The m6A contents of total RNAs in SN12-PM6 cell line. **m** P2RX6 mRNA and protein expression level after over-expression METTL14. M14 = METTL14, *N.S.* = not significant. * *P* < 0.05, ** *P* < 0.01, *** *P* < 0.001

Recent studies have shown that RNA methylation contributes to RCC development [20], so we sought to find out whether the high expression of P2RX6 in RCC was regulated by m^6^A methylation. To explore the potential mechanism of m^6^A modification in P2RX6, firstly, we predicted P2RX6 potential m^6^A sites with m^6^A Finder (http://m6a.renlab.org/) and found that P2RX6 has numbers of very high confidence m^6^A sites distribution (Fig. 5a, Additional file 6: Table S6). Then, with the colorimetric m^6^A quantification assay, we measured the levels of m^6^A in the total RNAs of 7 paired renal tumor tissues and adjacent tissues. We found that m^6^A levels were decreased in renal tumor tissues compared with their adjacent tissues (Fig. 5b). Recently, numbers of m^6^A modification related proteins have been identified, including NSUN2, FTO, METTL3, METTL14, ALKBH5 and YTHDF2 et al. [40]. We measured the expression levels of eleven m^6^A modification related genes in seven paired renal tumor tissues. We found that METTL14 and RBM15B mRNA expression significantly decreased, while FTO significantly increased in tumor tissues (Fig. 5c). Then, analyzing TCGA KIRC database, we found METTL14 mRNA was decreased in tumor tissues compared to normal tissues (Fig. 5d). Meanwhile, Kaplan-Meier analysis demonstrated lower METTL14 expression has worse OS result (***P < 0.0001) (Fig. 5e) (Fig. 5d-e obtained from UALCAN website http://ualcan.path.uab.edu/) [41]. Interesting, METTL14 has a negative correlation with P2RX6 gene in TCGA database (R^2^ = 0.089, ***P < 0.0001) (Fig. 5f) and website tool R2 (https://hgserver1.amc.nl/) (R^2^ = 0.099, ***P < 0.0046) (Fig. 5g, Additional file 7: Table S7). However, RBM15B has lower expression in tumor tissues and lower expression could result in worse OS, also RBM15B has a positive correlation with P2RX6 expression in TCGA database (Additional file 11: Figure S4 A-C). Meanwhile, FTO has higher expression in tumor tissues and a negative correlation with P2RX6 expression, but survival analysis has no significantly difference between two groups (Additional file 11: Figure S4 D-F). Combined with the above results, METTL14, an important m^6^A methyltransferase that has a positive correlation with m^6^A levels, was then considered as the candidate molecule for aberrant m^6^A modification in RCC.

Next, we implemented the interruption approaches using specific shRNA (sh-M14) to block METTL14 expression (Fig. 5h) and then examined m^6^A levels in the RCC cells. Consistently, knocking down METTL14 led to decrease m^6^A levels in 786-O cell (Fig. 5i) and increase P2RX6 mRNA and protein level (Fig. 5j). Furthermore, after over-expressing METTL14 (OE-M14) in SN12-PM6 cell line, the results are consistent with the knocking-down data (Fig. 5k-m).

Together, results from Fig. 5a-m suggested METTL14 might abrogate P2RX6 protein level via m^6^A methylated modification.

### Preclinical study using in vivo mouse model confirms that the ATP-P2RX6-Ca^2+^ −p-ERK1/2-MMP9 axis increases RCC metastasis

**Fig. 6:**
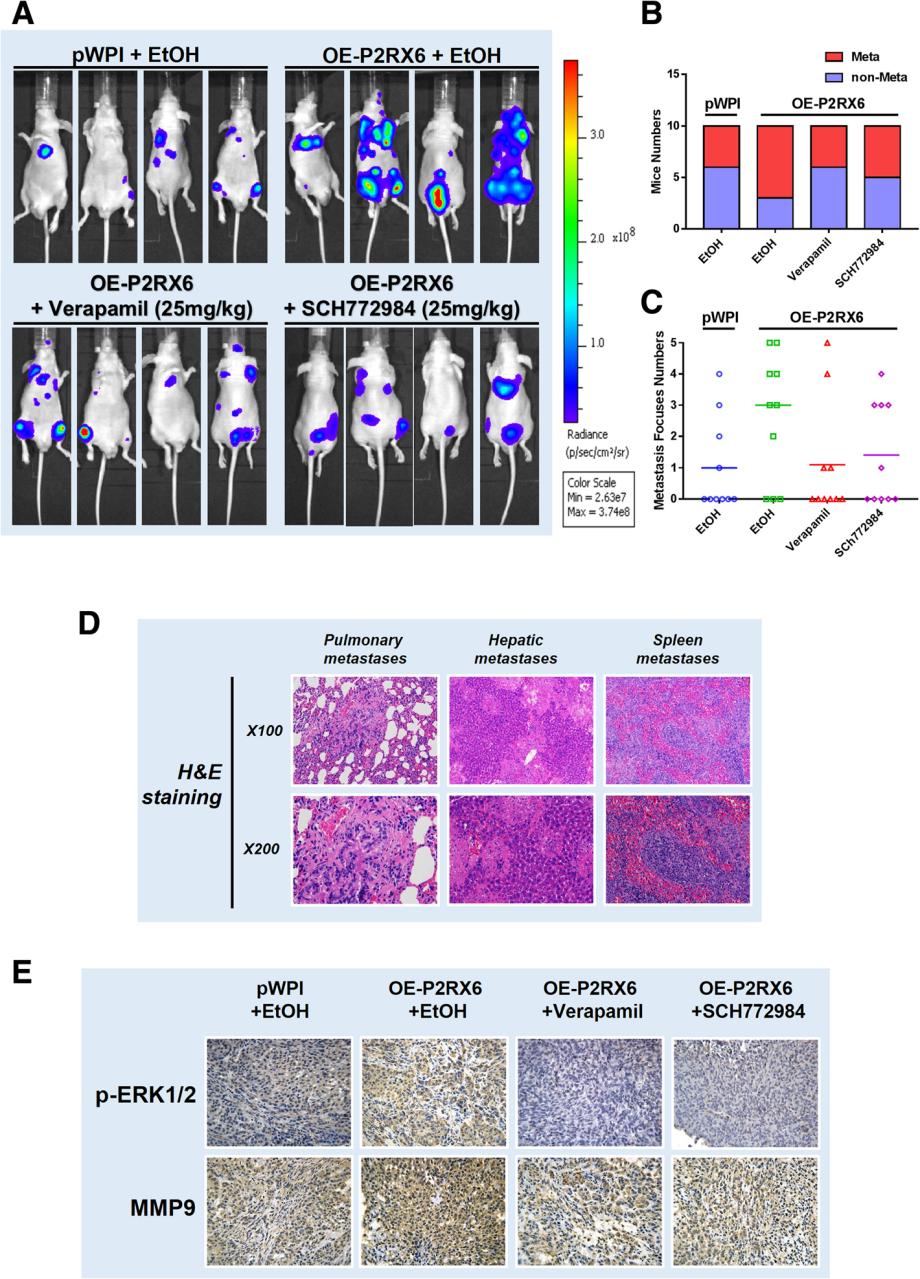
Preclinical study using mouse model to confirm ATP increased RCC metastasis via P2RX6-Ca^2+^ −p-ERK1/2-MMP9 axis. **a** The experimental scheme. The tumor metastases in nude mice implanted with OS-RC-2 cells. The nude mice were divided into 4 groups: pWPI-vector + EtOH (Mock), OE-P2RX6 + EtOH, OE-P2RX6 + Verapamil, OE-P2RX6 + SCH772984. Mice were sacrificed after 8 weeks were assessed for metastasis. The IVIS image for monitoring tumor and metastasis. **b** Quantitative analysis for Fig. 6a. **c** Number of metastasis foci in each groups. **d** Hematoxylin and eosin (H&E) staining were performed to confirm the tumor type. **e** Representative IHC images and quantification of p-ERK1/2 and MMP9 expression on mice metastasis foci

To confirm above in vitro cell lines data in the in vivo mouse model, we injected xenografted RCC OS-RC-2 cells expressing firefly luciferase into BALB/c nude mice tail vein [42]. After 8 weeks of implantation, the mice were sacrificed, the metastatic sites were further examined. The results indicated that mice received OE-P2RX6 injection saliently developed more metastatic tumors than the vehicle group (Fig. 6a-b). Importantly, using small molecules of Ca^2+^ influx (verapamil) or p-ERK1/2(SCH772984) to suppress the ATP-P2RX6-Ca^2+^ −p-ERK1/2 signaling all led to suppress the RCC progression and metastasis (Fig. 6c). In addition, anatomic studies were carried out and the histological staining were performed to confirm the tumor type (Fig. 6d).

IHC staining also testified that the expression of p-ERK1/2, MMP9, were higher in OE-P2RX6 group mice compared to the vehicle control, and using small molecules of Ca^2+^ influx or p-ERK1/2 to suppress the P2RX6-Ca^2+^ −p-ERK1/2 signaling all led to suppress those OE-P2RX6-increased p-ERK1/2-MMP9 signaling (Fig. 6e).

Together, preclinical study results from in vivo RCC mouse model (Fig. 6a-e) were in agreement with in vitro cell lines data illustrating ATP-OE-P2RX6 could enhance RCC metastasis via altering the ATP-P2RX6-Ca^2+^ p-ERK1/2-MMP9 signaling.

**Fig. 7:**
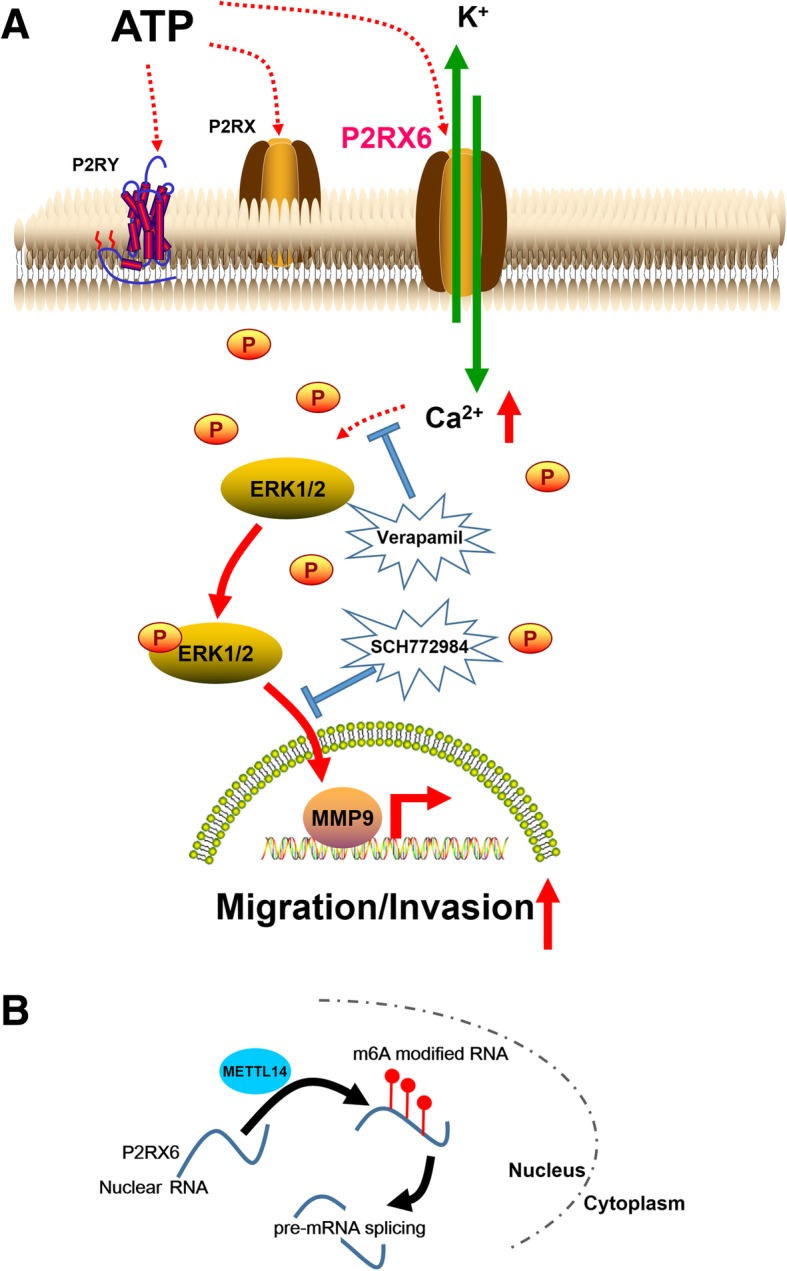
The cartoon model of METTL14-P2RX6-Ca^2+^ −p-ERK1/2-MMP9 axis signal on RCC cell migration and invasion. **a** P2RX6’s specific mechanism on metastasis. **b** METTL14’s specific mechanism on regulation P2RX6 mRNA m^6^A methylation

As summarized in Fig. 7a-b, the m6A-suppressed P2RX6 activation promotes renal cancer cells migration and invasion through ATP-induced Ca^2+^ influx modulating ERK1/2 phosphorylation and MMP9 signalling pathway.

## Discussion

A number of studies have found that tumor microenvironment extracellular ATP might play a detrimental role in tumor progression [9, 10, 43] and initiate signaling pathways through activating membrane receptors. Previous reports demonstrated that extracellular ATP activates P2RY2 and promotes prostate cancer cells invasion and metastasis [44]. Li et al. revealed that ATP or UTP could activate EGFR and ERK1/2 and P2RY2 could suppress ATP-induced phosphorylation of EGFR and ERK1/2. Besides, among P2RX receptors, P2RX7 draw many attentions. P2RX7 activation releases important pro-inflammatory cytokines such as interleukin-1β and fever-inducing prostaglandin E2, which is crucial for initiation of the inflammatory signaling cascade [31, 45–47]. As such, studies have recently shown that P2RX7 participated in tumors metastasis, and it is up-regulated compared with normal tissues, including chronic lymphocyte leukemia, melanoma, neuroblastoma, prostate, breast and thyroid cancers [13, 33, 48, 49].

In recent years, P2RX6 function has been researched widely [50, 51]. In transiently transfected HEK293 cell, it has been reported that the recombinant homomeric P2RX6 complexes reach the plasma membrane. There are also researchers proved that P2RX6 can only play a role at the cell surface in the synergy of P2X4 or P2X2 [52, 53]. Our preliminary data showed that ATP could promote RCC migration and invasion and P2RX6 might play a crucial role according to bioinformatics analysis. Till now, there is no report about P2RX6 specific mechanism in RCC metastasis.

In this study, we found membrane protein P2RX6, whose higher expression could be detected in several RCC cells compared with HK2 normal cell. Clinical survey and TCGA database analysis performed that P2RX6 expression was obviously correlated with RCC pathological stage, pathological grade and organ metastasis. We therefore focused on P2RX6 for further analysis. Preclinical studies using in vitro multiple RCC cells and in vivo mouse model all demonstrated P2RX6 might function via ATP-P2RX6-Ca^2+^ −p-ERK1/2-MMP9 axis to drive RCC cells invasion and metastasis.

As one of the most important second messengers in intracellular signaling network, Ca^2+^ flux and Ca^2+^-dependent signaling also played a pivotal role in cancer progression [54, 55]. Upon initiation of the Ca^2+^ ion channel and phosphorylation of its substrate, an intracellular calcium signaling cascade is activated that mediates a variety of biological processes by coordinating with other abnormal signals and modulating the activity of subsequent transcription factors [56–58]. This study illustrated that verapamil could block the Ca^2+^ influx, which is caused by P2RX6. Elucidation of this mechanism facilitates the identification of new RCC biomarkers and helps in the development of new targeted therapies.

Previous studies reported that ERK can be activated by Ca^2+^ and then phosphorylates and activates various transcription factors and other molecular effectors [59]. Here, we found that Ca^2+^ signal as elicited by ATP via P2RX6 caused ERK1/2 phosphorylation. Indeed, application of 0.1uM SCH772984, a concentration that had no effect on ERK1/2 phosphorylation, could then blunt the ATP-enhanced RCC cell migration and invasion. This result demonstrated that the P2RX6 signal and the Ca^2+^ signal synergistically induced ERK1/2 phosphorylation.

So far, studies have shown that a large number of m^6^A-related enzymes play a key role in tumor development. These enzymes are mainly divided into two categories, one is methyltransferase (METTL3/14/16, WTAP, KIAA1429 and RBM15/15B et al) and the other is demethylase (FTO and ALKBH5 et al) [20]. Interestingly, with screening the potential m^6^A candidates, we identified that METTL14 could suppress P2RX6 mRNA and protein level. That is to say, METTL14 may increase P2RX6 pre-mRNA splicing by mediating P2RX6 mRNA level m^6^A methylation. The specific mechanism is not clear, but it provides a direction for subsequent research.

## Conclusions

In conclusion, our preclinical studies using multiple in vitro cell lines and in vivo mouse models as well as human clinical studies all suggest that ATP-P2RX6-Ca^2+^ −p-ERK1/2-MMP9 axis facilitate RCC migration and invasion. The involvement of purine receptors in cancer cell biology is a promising area of research that can increase understanding of the processes that lead to cancer metastasis. This may lead to the development of new drug approaches in RCC treatment.

## Additional files

Additional file 1: **Table S1.** Oligonucleotide Sequences, Antibody and Inhibitor used in this study. (PPTX 62 kb) (PPTX 61 kb)

Additional file 2: **Table S2.** TCGA Clinical data. (XLSX 59 kb) (XLSX 58 kb)

Additional file 3: **Table S3.** Candidates selecting process & GO analysis result. (XLSX 49 kb)

Additional file 4: **Table S4.** P2RX6 IHC analysis of tissues microarray. (XLSX 10 kb) (XLSX 9 kb)

Additional file 5: **Table S5.** Clinical characteristics of 238 RCC patients according to P2RX6 expression levels. (PPTX 43 kb) (PPTX 42 kb)

Additional file 6: **Table S6.** P2RX6 m^6^A in plain text from. (XLSX 15 kb) (XLSX 14 kb)

Additional file 7: **Table S7.** Correlation between METTL14 and P2RX6. (XLSX 12 kb) (XLSX 11 kb)

Additional file 8: **Figure S1.** Candidates selecting and P2RX6 gene bioinformatics characteristic. A P2RX6 gene selecting process flowchart. B ADRA2A gene expression in different stages. C Kaplan-Meier analysis for ADRA2A mRNA expression in RCC patients. D P2RX6 gene information on http://www.oncolnc.org/ and Kaplan-Meier analysis for P2RX6 mRNA expression in RCC patients (**P = 0.00175). (TIF 1943 kb)

Additional file 9: **Figure S2.** ATP effects on migration/invasion of OS-RC-2 cells and validating P2RX6 gene function in SW839 cell line. A In vitro wound-healing motility assay with 3, 10, 30uM ATP treatment in OS-RC-2 cells. B Transwell invasion assays were performed with 3, 10, 30uM ATP treatment in OS-RC-2 cells. C Quantitative analysis for Fig. S1B. D qRT-PCR assay validation of P2RX6 mRNA level knocking-down efficiency and E WB validation of P2RX6 protein level knocking-down efficiency when knocking down using sh-P2RX6^#1^ in SW839 cells. F Migration assay after using sh-P2RX6^#1^ in SW839 cells treated with ATP, PLKO.1-vector as control. G Quantitative analysis for Fig. S2 F.H Transwell assay were performed after using sh-P2RX6^#1^ in SW839 cells treated with ATP, PLKO.1-vector as control. I Quantitative analysis for Fig. S2 H. * indicated *P* < 0.05. And ** indicated *P* < 0.01, *** indicated *P* < 0.001. (TIF 2967 kb)

Additional file 10: **Figure S3.** Excluding MMP13 as downstream target gene. A Correlation analysis for P2RX6 and MMP13 from TCGA database. B WB validation of MMP13 protein level after treating with ATP. (TIF 786 kb)

Additional file 11: **Figure S4.** Excluding RBM15B and FTO as target methylated gene. A RBM15B has lower expression in tumor tissues and B lower expression could result in a worse OS, and C has a positive correlation with P2RX6 expression in TCGA database. D FTO has higher expression in tumor tissues and E survival analysis has no significantly difference between two groups. F FTO has a negative correlation with P2RX6 expression (TIF 939 kb)

## Abbreviations

RCC: Renal cell carcinoma
m^6^A: N6-methyladenosine
ATP: Adenosine 5′-triphosphate
qRT-PCR: quantitative real-time PCR analysis
WB: Western immunoblotting
IF: Immunofluorescence
IHC: Immunohistochemical
