# Supplementary material for: The m6A-suppressed P2RX6 activation promotes renal cancer cells migration and invasion through ATP-induced Ca2+ influx modulating ERK1/2 phosphorylation and MMP9 signaling pathway
Source: J Exp Clin Cancer Res. 2019 Jun 3;38:233. doi: 10.1186/s13046-019-1223-y (PMC6547495; doi:10.1186/s13046-019-1223-y)
Supplement: Supplementary file 1 — Table S1. Oligonucleotide Sequences, Antibody and Inhibitor used in this study. (PPTX 62 kb) (PPTX 61 kb) [file 13046_2019_1223_MOESM1_ESM.pptx]

## Slide 1
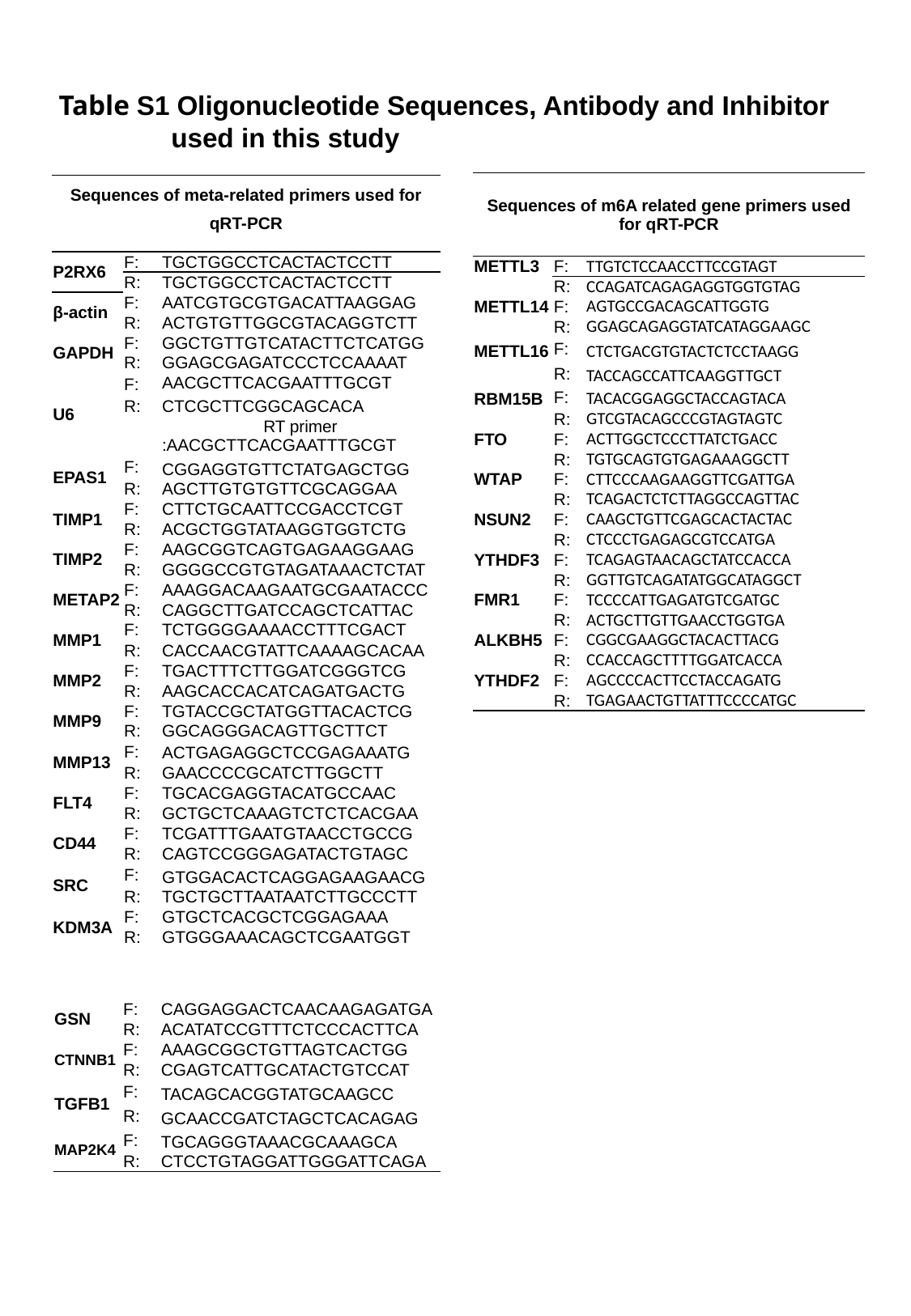

Table S1 Oligonucleotide Sequences, Antibody and Inhibitor
 used in this study
| Sequences of m6A related gene primers used for qRT-PCR | | |
| --- | --- | --- |
| METTL3 | F: | TTGTCTCCAACCTTCCGTAGT |
| | R: | CCAGATCAGAGAGGTGGTGTAG |
| METTL14 | F: | AGTGCCGACAGCATTGGTG |
| | R: | GGAGCAGAGGTATCATAGGAAGC |
| METTL16 | F: | CTCTGACGTGTACTCTCCTAAGG |
| | R: | TACCAGCCATTCAAGGTTGCT |
| RBM15B | F: | TACACGGAGGCTACCAGTACA |
| | R: | GTCGTACAGCCCGTAGTAGTC |
| FTO | F: | ACTTGGCTCCCTTATCTGACC |
| | R: | TGTGCAGTGTGAGAAAGGCTT |
| WTAP | F: | CTTCCCAAGAAGGTTCGATTGA |
| | R: | TCAGACTCTCTTAGGCCAGTTAC |
| NSUN2 | F: | CAAGCTGTTCGAGCACTACTAC |
| | R: | CTCCCTGAGAGCGTCCATGA |
| YTHDF3 | F: | TCAGAGTAACAGCTATCCACCA |
| | R: | GGTTGTCAGATATGGCATAGGCT |
| FMR1 | F: | TCCCCATTGAGATGTCGATGC |
| | R: | ACTGCTTGTTGAACCTGGTGA |
| ALKBH5 | F: | CGGCGAAGGCTACACTTACG |
| | R: | CCACCAGCTTTTGGATCACCA |
| YTHDF2 | F: | AGCCCCACTTCCTACCAGATG |
| | R: | TGAGAACTGTTATTTCCCCATGC |
| Sequences of meta-related primers used for qRT-PCR | | |
| --- | --- | --- |
| P2RX6 | F: | TGCTGGCCTCACTACTCCTT |
| | R: | TGCTGGCCTCACTACTCCTT |
| β-actin | F: | AATCGTGCGTGACATTAAGGAG |
| | R: | ACTGTGTTGGCGTACAGGTCTT |
| GAPDH | F: | GGCTGTTGTCATACTTCTCATGG |
| | R: | GGAGCGAGATCCCTCCAAAAT |
| U6 | F: | AACGCTTCACGAATTTGCGT |
| | R: | CTCGCTTCGGCAGCACA |
| | | RT primer :AACGCTTCACGAATTTGCGT |
| EPAS1 | F: | CGGAGGTGTTCTATGAGCTGG |
| | R: | AGCTTGTGTGTTCGCAGGAA |
| TIMP1 | F: | CTTCTGCAATTCCGACCTCGT |
| | R: | ACGCTGGTATAAGGTGGTCTG |
| TIMP2 | F: | AAGCGGTCAGTGAGAAGGAAG |
| | R: | GGGGCCGTGTAGATAAACTCTAT |
| METAP2 | F: | AAAGGACAAGAATGCGAATACCC |
| | R: | CAGGCTTGATCCAGCTCATTAC |
| MMP1 | F: | TCTGGGGAAAACCTTTCGACT |
| | R: | CACCAACGTATTCAAAAGCACAA |
| MMP2 | F: | TGACTTTCTTGGATCGGGTCG |
| | R: | AAGCACCACATCAGATGACTG |
| MMP9 | F: | TGTACCGCTATGGTTACACTCG |
| | R: | GGCAGGGACAGTTGCTTCT |
| MMP13 | F: | ACTGAGAGGCTCCGAGAAATG |
| | R: | GAACCCCGCATCTTGGCTT |
| FLT4 | F: | TGCACGAGGTACATGCCAAC |
| | R: | GCTGCTCAAAGTCTCTCACGAA |
| CD44 | F: | TCGATTTGAATGTAACCTGCCG |
| | R: | CAGTCCGGGAGATACTGTAGC |
| SRC | F: | GTGGACACTCAGGAGAAGAACG |
| | R: | TGCTGCTTAATAATCTTGCCCTT |
| KDM3A | F: | GTGCTCACGCTCGGAGAAA |
| | R: | GTGGGAAACAGCTCGAATGGT |
| GSN | F: | CAGGAGGACTCAACAAGAGATGA |
| --- | --- | --- |
| | R: | ACATATCCGTTTCTCCCACTTCA |
| CTNNB1 | F: | AAAGCGGCTGTTAGTCACTGG |
| | R: | CGAGTCATTGCATACTGTCCAT |
| TGFB1 | F: | TACAGCACGGTATGCAAGCC |
| | R: | GCAACCGATCTAGCTCACAGAG |
| MAP2K4 | F: | TGCAGGGTAAACGCAAAGCA |
| | R: | CTCCTGTAGGATTGGGATTCAGA |

## Slide 2
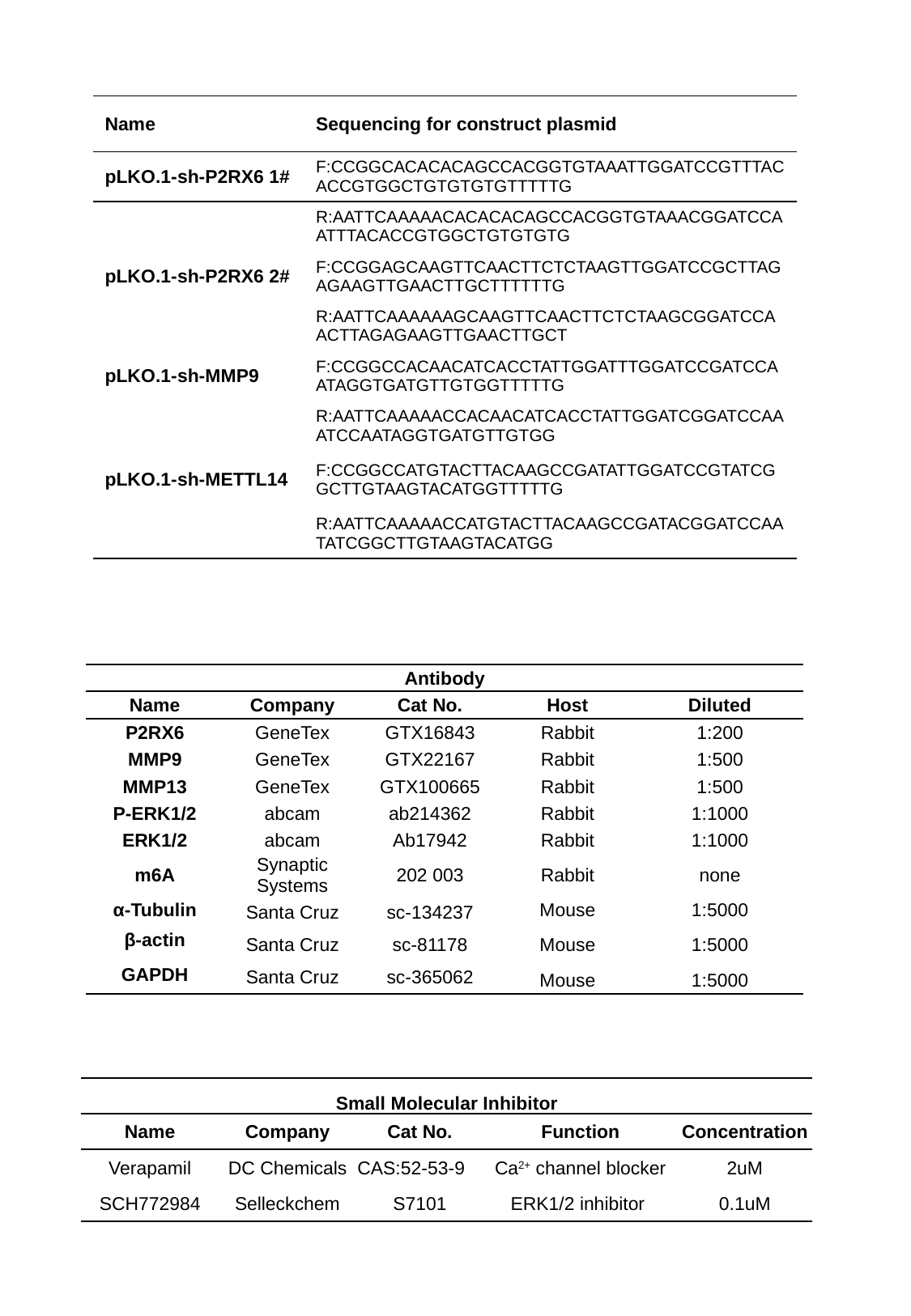

| Name | Sequencing for construct plasmid |
| --- | --- |
| pLKO.1-sh-P2RX6 1# | F:CCGGCACACACAGCCACGGTGTAAATTGGATCCGTTTACACCGTGGCTGTGTGTGTTTTTG |
| | R:AATTCAAAAACACACACAGCCACGGTGTAAACGGATCCAATTTACACCGTGGCTGTGTGTG |
| pLKO.1-sh-P2RX6 2# | F:CCGGAGCAAGTTCAACTTCTCTAAGTTGGATCCGCTTAGAGAAGTTGAACTTGCTTTTTTG |
| | R:AATTCAAAAAAGCAAGTTCAACTTCTCTAAGCGGATCCAACTTAGAGAAGTTGAACTTGCT |
| pLKO.1-sh-MMP9 | F:CCGGCCACAACATCACCTATTGGATTTGGATCCGATCCAATAGGTGATGTTGTGGTTTTTG |
| | R:AATTCAAAAACCACAACATCACCTATTGGATCGGATCCAAATCCAATAGGTGATGTTGTGG |
| pLKO.1-sh-METTL14 | F:CCGGCCATGTACTTACAAGCCGATATTGGATCCGTATCGGCTTGTAAGTACATGGTTTTTG |
| | R:AATTCAAAAACCATGTACTTACAAGCCGATACGGATCCAATATCGGCTTGTAAGTACATGG |
| Antibody | | | | |
| --- | --- | --- | --- | --- |
| Name | Company | Cat No. | Host | Diluted |
| P2RX6 | GeneTex | GTX16843 | Rabbit | 1:200 |
| MMP9 | GeneTex | GTX22167 | Rabbit | 1:500 |
| MMP13 | GeneTex | GTX100665 | Rabbit | 1:500 |
| P-ERK1/2 | abcam | ab214362 | Rabbit | 1:1000 |
| ERK1/2 | abcam | Ab17942 | Rabbit | 1:1000 |
| m6A | Synaptic Systems | 202 003 | Rabbit | none |
| α-Tubulin | Santa Cruz | sc-134237 | Mouse | 1:5000 |
| β-actin | Santa Cruz | sc-81178 | Mouse | 1:5000 |
| GAPDH | Santa Cruz | sc-365062 | Mouse | 1:5000 |
| Small Molecular Inhibitor | | | | |
| --- | --- | --- | --- | --- |
| Name | Company | Cat No. | Function | Concentration |
| Verapamil | DC Chemicals | CAS:52-53-9 | Ca2+ channel blocker | 2uM |
| SCH772984 | Selleckchem | S7101 | ERK1/2 inhibitor | 0.1uM |
