# Supplementary material for: The m6A-suppressed P2RX6 activation promotes renal cancer cells migration and invasion through ATP-induced Ca2+ influx modulating ERK1/2 phosphorylation and MMP9 signaling pathway
Source: J Exp Clin Cancer Res. 2019 Jun 3;38:233. doi: 10.1186/s13046-019-1223-y (PMC6547495; doi:10.1186/s13046-019-1223-y)
Supplement: Supplementary file 5 — Table S5. Clinical characteristics of 238 RCC patients according to P2RX6 expression levels. (PPTX 43 kb) (PPTX 42 kb) [file 13046_2019_1223_MOESM5_ESM.pptx]

## Slide 1
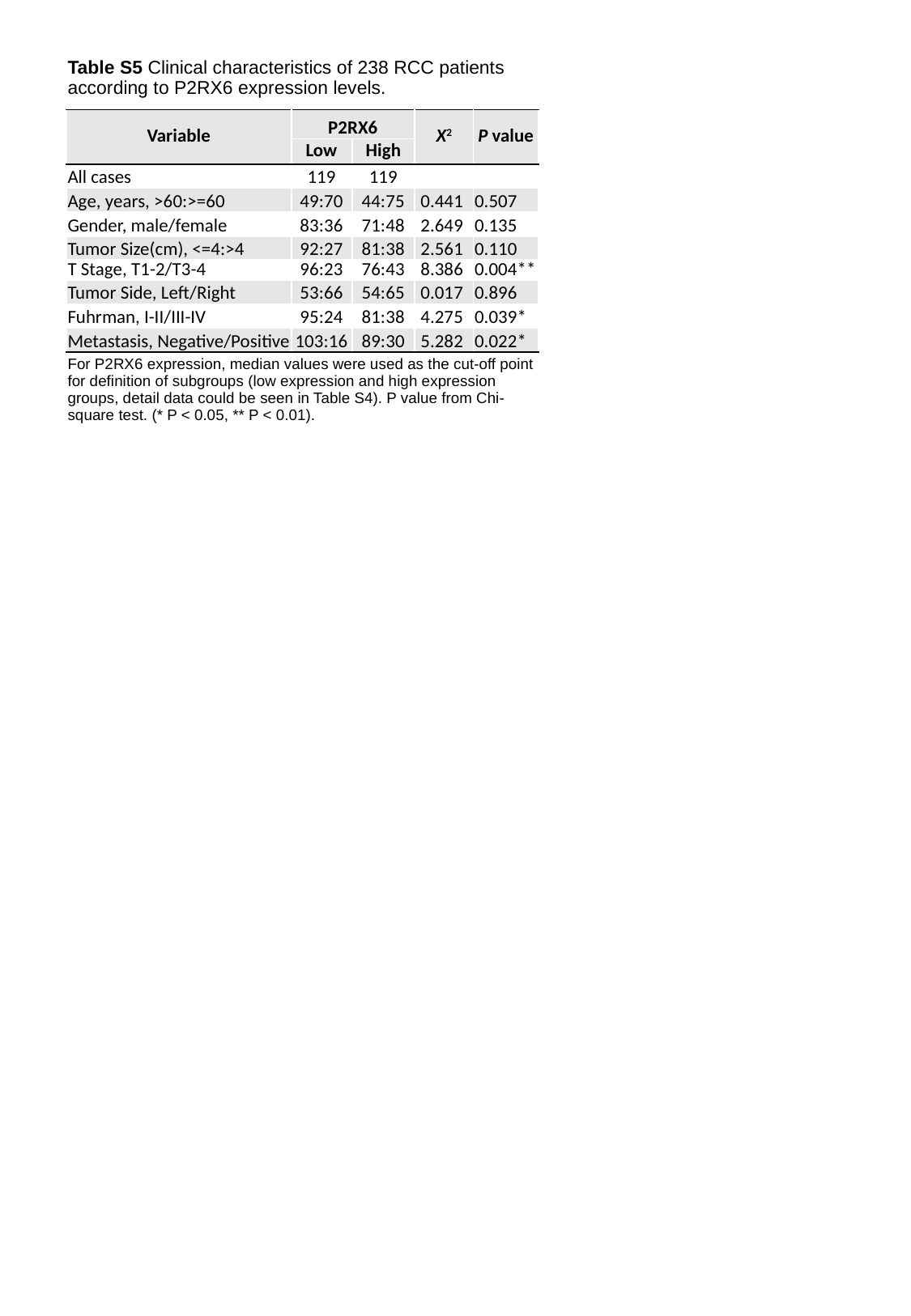

| Table S5 Clinical characteristics of 238 RCC patients according to P2RX6 expression levels. | | | | |
| --- | --- | --- | --- | --- |
| Variable | P2RX6 | | X2 | P value |
| | Low | High | | |
| All cases | 119 | 119 | | |
| Age, years, >60:>=60 | 49:70 | 44:75 | 0.441 | 0.507 |
| Gender, male/female | 83:36 | 71:48 | 2.649 | 0.135 |
| Tumor Size(cm), <=4:>4 | 92:27 | 81:38 | 2.561 | 0.110 |
| T Stage, T1-2/T3-4 | 96:23 | 76:43 | 8.386 | 0.004\*\* |
| Tumor Side, Left/Right | 53:66 | 54:65 | 0.017 | 0.896 |
| Fuhrman, I-II/III-IV | 95:24 | 81:38 | 4.275 | 0.039\* |
| Metastasis, Negative/Positive | 103:16 | 89:30 | 5.282 | 0.022\* |
| For P2RX6 expression, median values were used as the cut-off point for definition of subgroups (low expression and high expression groups, detail data could be seen in Table S4). P value from Chi-square test. (\* P < 0.05, \*\* P < 0.01). | | | | |
