# Supplementary figures and images for: The m6A-suppressed P2RX6 activation promotes renal cancer cells migration and invasion through ATP-induced Ca2+ influx modulating ERK1/2 phosphorylation and MMP9 signaling pathway
Source: J Exp Clin Cancer Res. 2019 Jun 3;38:233. doi: 10.1186/s13046-019-1223-y (PMC6547495; doi:10.1186/s13046-019-1223-y)

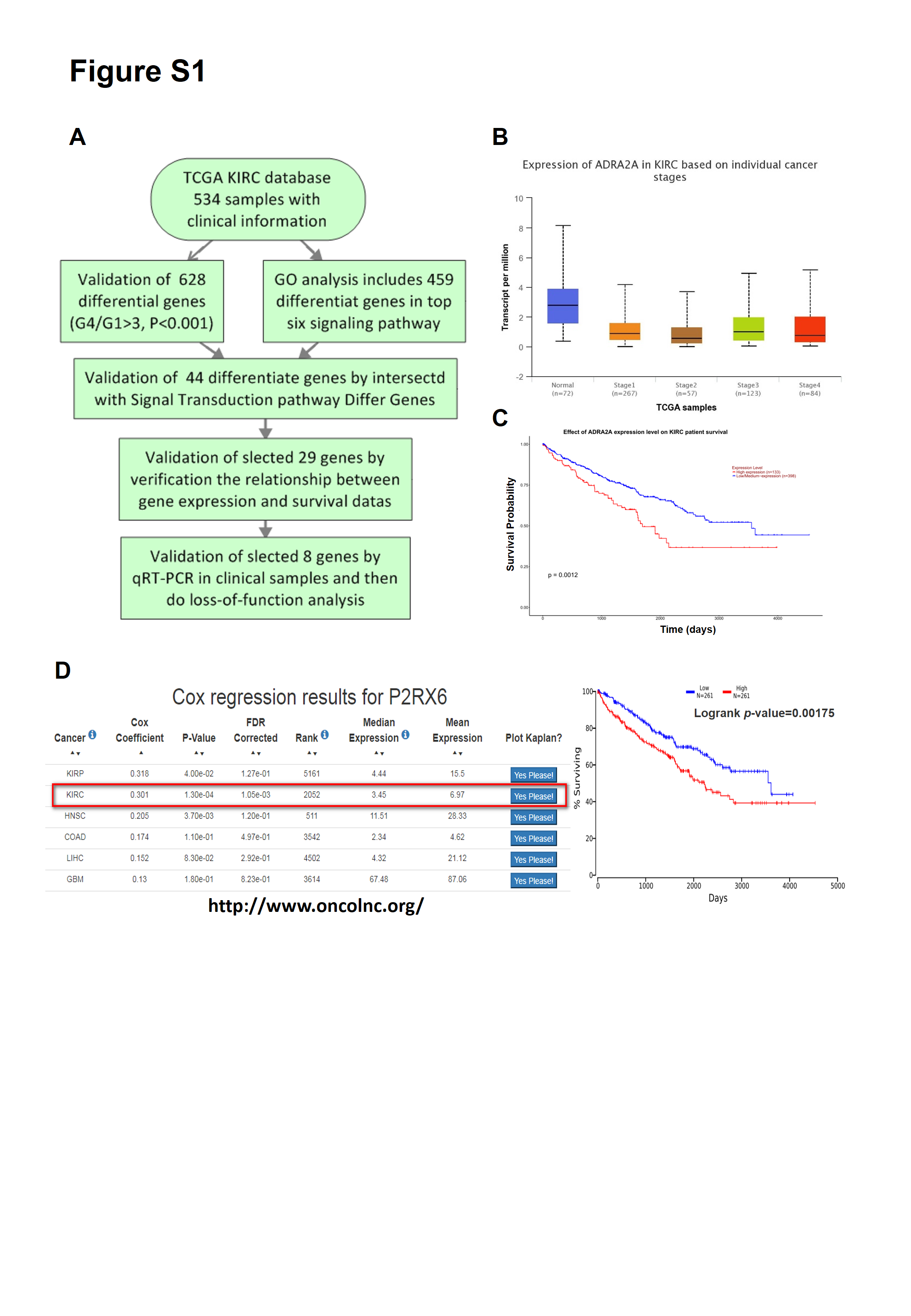

Supplement: Supplementary file 8 — Figure S1. Candidates selecting and P2RX6 gene bioinformatics characteristic. A P2RX6 gene selecting process flowchart. B ADRA2A gene expression in different stages. C Kaplan-Meier analysis for ADRA2A mRNA expression in RCC patients. D P2RX6 gene information on http://www.oncolnc.org/ and Kaplan-Meier analysis for P2RX6 mRNA expression in RCC patients (**P = 0.00175). (TIF 1943 kb) [file 13046_2019_1223_MOESM8_ESM.tif]

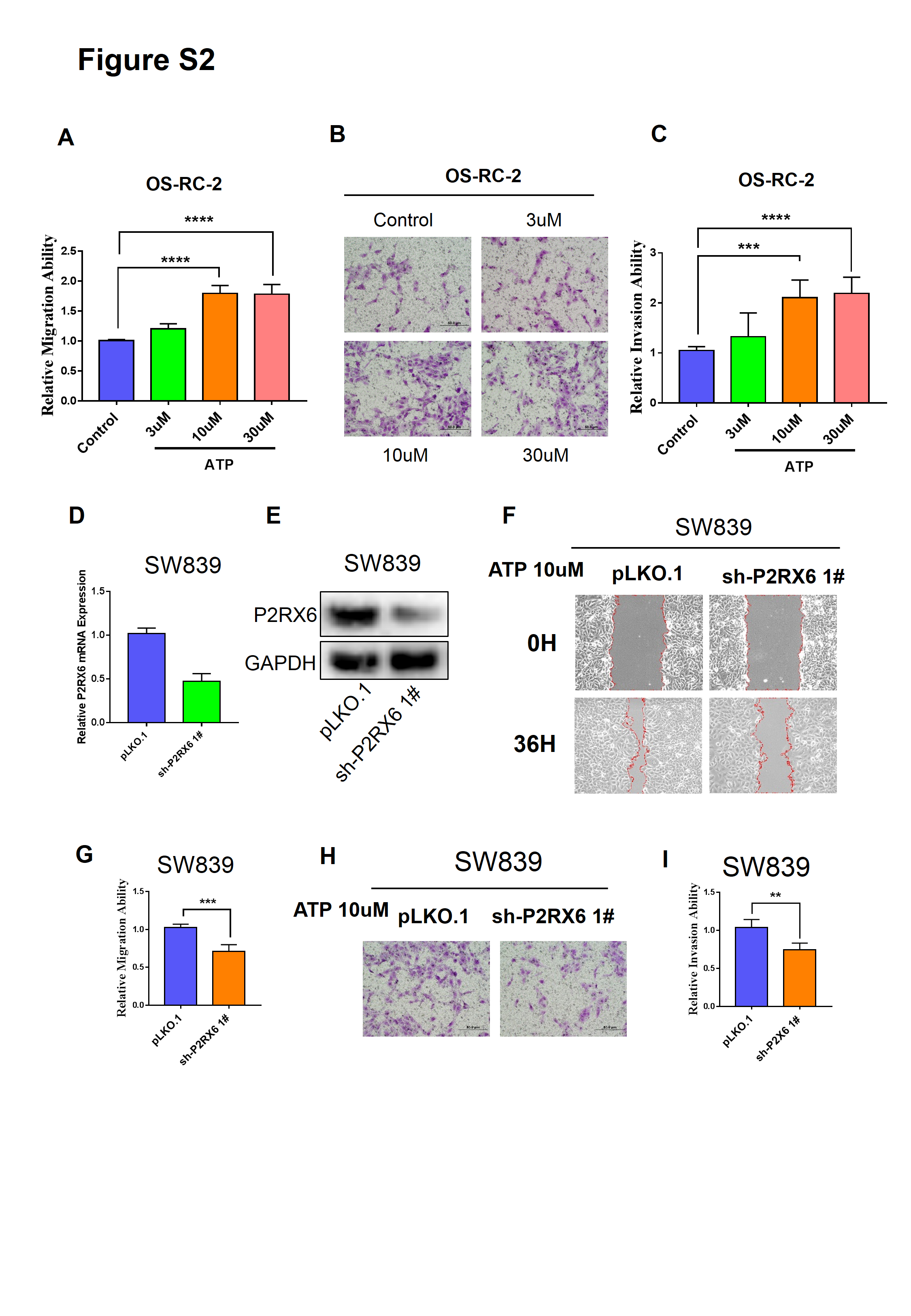

Supplement: Supplementary file 9 — Figure S2. ATP effects on migration/invasion of OS-RC-2 cells and validating P2RX6 gene function in SW839 cell line. A In vitro wound-healing motility assay with 3, 10, 30uM ATP treatment in OS-RC-2 cells. B Transwell invasion assays were performed with 3, 10, 30uM ATP treatment in OS-RC-2 cells. C Quantitative analysis for Fig. S1B. D qRT-PCR assay validation of P2RX6 mRNA level knocking-down efficiency and E WB validation of P2RX6 protein level knocking-down efficiency when knocking down using sh-P2RX6#1 in SW839 cells. F Migration assay after using sh-P2RX6#1 in SW839 cells treated with ATP, PLKO.1-vector as control. G Quantitative analysis for Fig. S2 F.H Transwell assay were performed after using sh-P2RX6#1 in SW839 cells treated with ATP, PLKO.1-vector as control. I Quantitative analysis for Fig. S2 H. * indicated P < 0.05. And ** indicated P < 0.01, *** indicated P < 0.001. (TIF 2967 kb) [file 13046_2019_1223_MOESM9_ESM.tif]

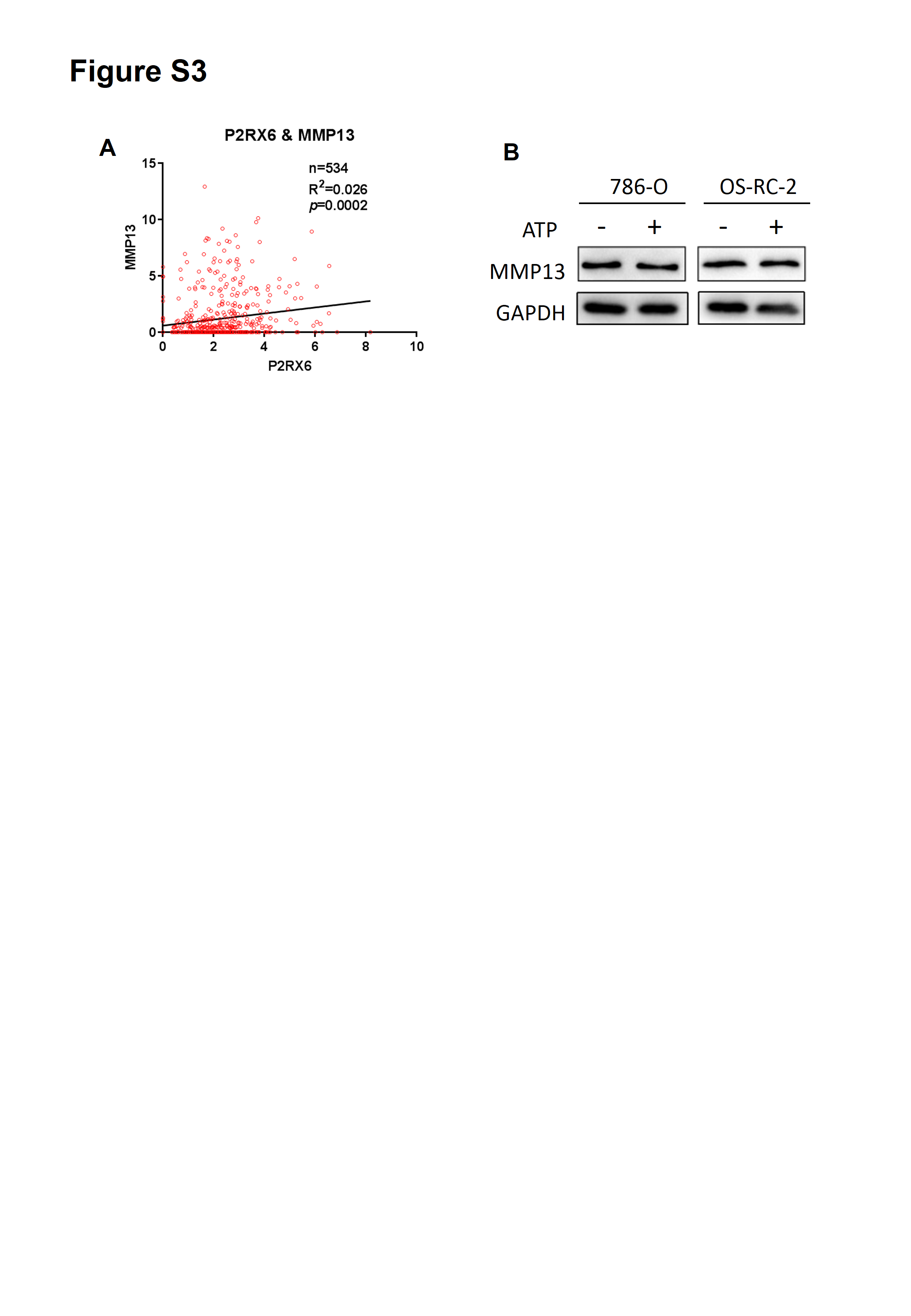

Supplement: Supplementary file 10 — Figure S3. Excluding MMP13 as downstream target gene. A Correlation analysis for P2RX6 and MMP13 from TCGA database. B WB validation of MMP13 protein level after treating with ATP. (TIF 786 kb) [file 13046_2019_1223_MOESM10_ESM.tif]

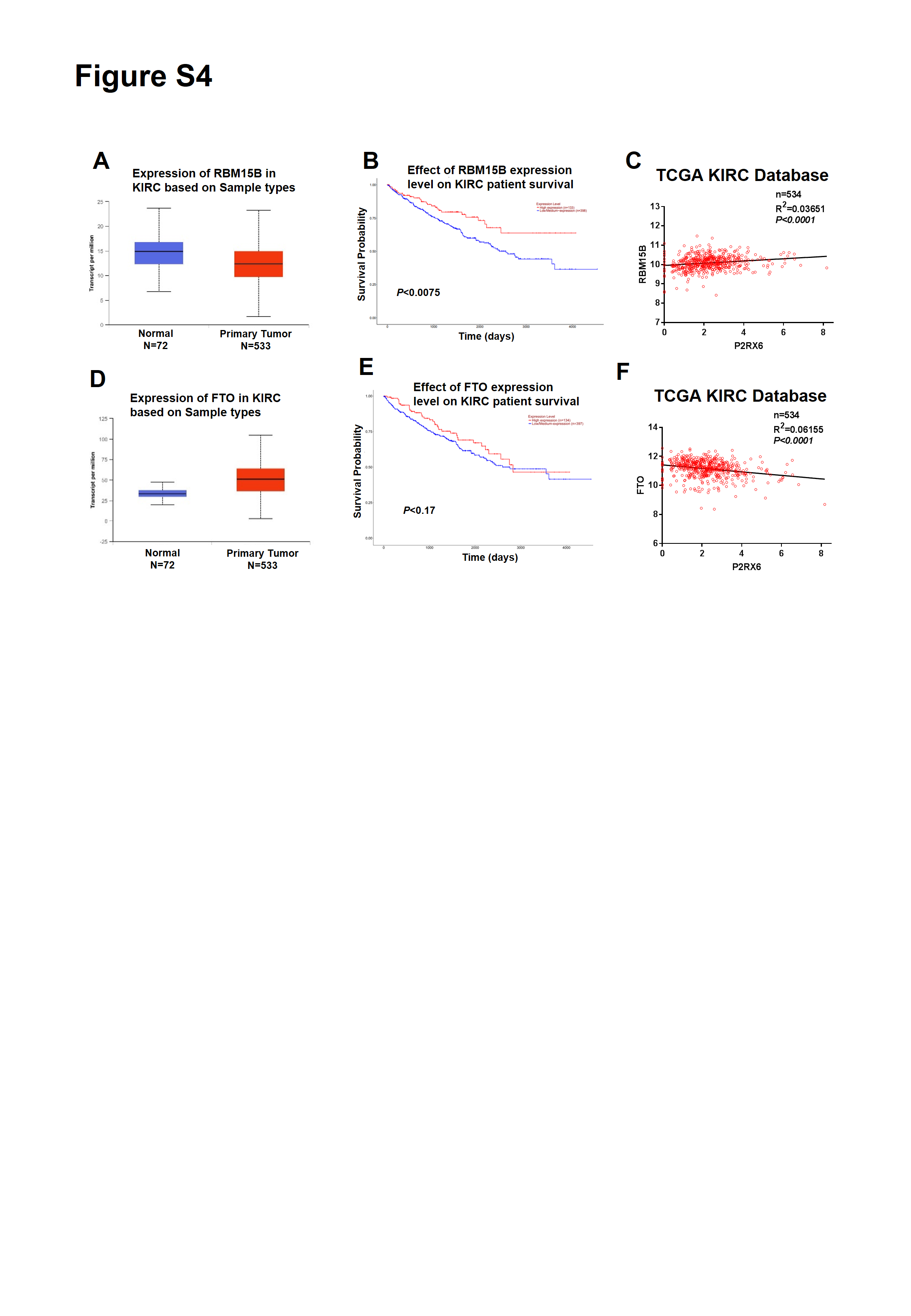

Supplement: Supplementary file 11 — Figure S4. Excluding RBM15B and FTO as target methylated gene. A RBM15B has lower expression in tumor tissues and B lower expression could result in a worse OS, and C has a positive correlation with P2RX6 expression in TCGA database. D FTO has higher expression in tumor tissues and E survival analysis has no significantly difference between two groups. F FTO has a negative correlation with P2RX6 expression (TIF 939 kb) [file 13046_2019_1223_MOESM11_ESM.tif]
